# A Versatile Method to Determine the Cellular Bioavailability of Small-Molecule Inhibitors

**DOI:** 10.1021/acs.jmedchem.6b00923

**Published:** 2017-01-03

**Authors:** Kevin B. Teuscher, Min Zhang, Haitao Ji

**Affiliations:** † Drug Discovery Department, H. Lee Moffitt Cancer Center and Research Institute, 12902 Magnolia Drive, Tampa, Florida 33612-9416, United States; ‡ Department of Oncologic Sciences, University of South Florida College of Medicine, Tampa, Florida 33612, United States; § Department of Chemistry, University of Utah, Salt Lake City, Utah 84112, United States

## Abstract

The determination of the cellular bioavailability of small-molecule inhibitors is a critical step for interpreting cell-based data and guiding inhibitor optimization. Herein, a HPLC-MS based protocol was developed to determine inhibitor cellular bioavailability. This generalizable protocol allows determination of the accurate intracellular concentrations and characterization of various properties of inhibitors including the extra- and intracellular stability, the dose- and time-dependence of the intracellular concentrations, the cell permeability, and the nonspecific binding with the cell culture plates, the extracellular matrices, and the cell membrane. The inhibitors of the protein–protein interactions, bromodomains, and the *β*-catenin/B-cell lymphoma 9 (BCL9) interaction were used to examine the protocol, and the cellular bioavailability of the inhibitors in cancer cells was determined. High nonspecific binding and low cellular uptake were observed for two bromodomain inhibitors. The two *β*-catenin/BCL9 inhibitors had low nonspecific binding but different cellular uptake. These inhibitors exhibited different stability kinetics in cells.

## Graphical Abstract

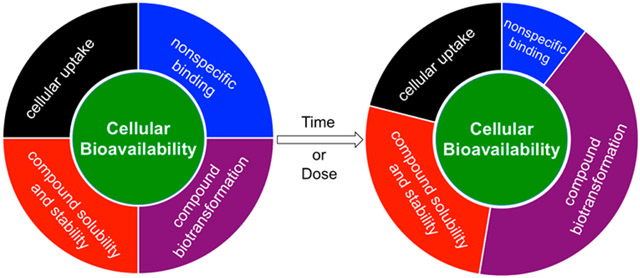

## INTRODUCTION

Selective small-molecule inhibitors for protein–protein interactions (PPIs) play an important role in facilitating the functional annotation of human genome and validating new molecular targets for therapeutic exploitation. These inhibitors can also serve as pathfinder molecules for intracellular signaling cascades and inform identification of useful biomarkers.^1,2^ The mismatches between biochemical and cell-based assay results have been a recurring problem when the new inhibitor identified from the biochemical studies is subjected to cell-based studies.^3-5^ This difference has traditionally been ascribed to the low permeability of the compound through the cell membrane. However, the cell-based data of small molecules can also be affected by compound aqueous solubility, the nonspecific binding with the serum proteins in the culture media, the culture plate, the extracellular matrices, and the cell membrane and the biotransformation to an inactive or active metabolite. Further, the cell-based results are dependent upon the time points when the data is collected. Hence, it is important to determine the cellular bioavailability for the correct interpretation of cell-based chemical biology data.^6-8^ Unfortunately, very few cellular bioavailability studies with the specialized technique have been reported.^9^ The field has lacked a protocol that is robust, sensitive, and generalizable and can be routinely used in chemical biology and medicinal chemistry laboratories. In this study, we detailed such a protocol to determine the cellular bioavailability of small-molecule inhibitors. This protocol has successfully been used to characterize the inhibitors for bromodomain-containing proteins, the epigenetic readers of histone lysine acetylation,^10,11^ and for the *β*-catenin/B-cell lymphoma 9 (BCL9) PPI, a key downstream effector of the canonical Wnt signaling pathway.^12^

## METHODS

**Figure 1.**
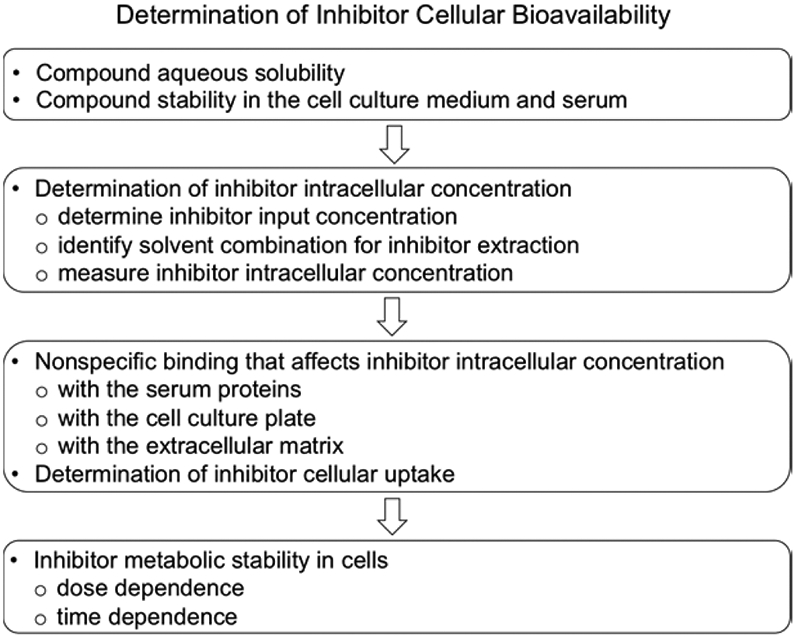
Workflow for determination of inhibitor cellular bioavailability.

**Figure 2.**
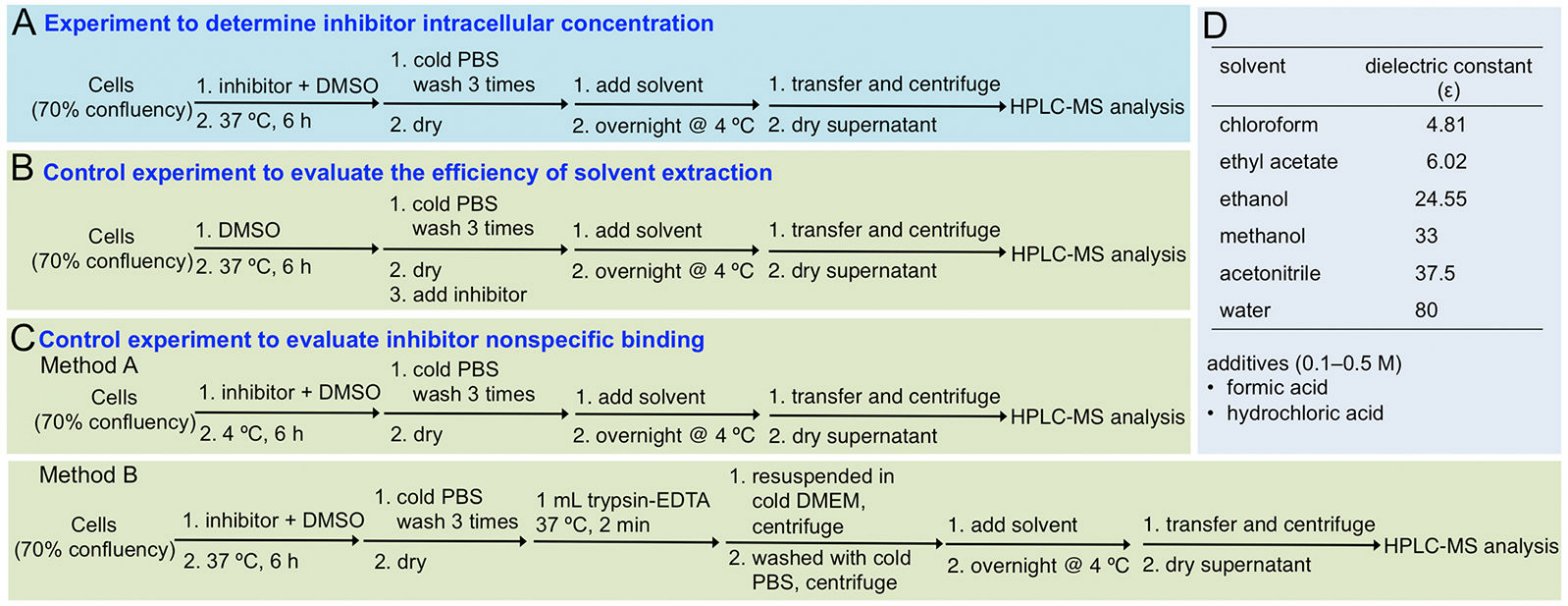
(A–C) The workflow for determination of inhibitor intracellular concentration and the workflows for two control experiments that evaluate the efficiency of solvent extraction and compound nonspecific binding. (D) Common solvents and additives used to extract small-molecule inhibitors from the studied cells.

The workflow for determination of inhibitor cellular bioavailability is shown in Figure 1. The first step is the assessment of compound physicochemical properties that include compound aqueous solubility and compound stability in cell culture medium and serum. The HPLC calibration curves also need to be determined for the tested inhibitors. The quantification of inhibitor intracellular concentration is the key step of this workflow. To achieve this goal, the input concentration of the tested compound will typically be set lower but close to the IC_50_ values of cell-based assay results. The protocol for determination of inhibitor intracellular concentration is shown in Figure 2A. The successful extraction of the inhibitor from the studied cells is critical for this study. The extraction efficiency of the applied solvent is determined using the control experiment in Figure 2B and is used to assess the optimal solvent combination for the quantification of inhibitor intracellular concentration. Figure 2D lists the common solvents that can be used to extract small-molecule inhibitors from cells. Organic solvents with lower hydrophobicity such as acetonitrile (MeCN)^13^ and methanol (MeOH)^14^ favor inactivating and breaking down the cell membrane to release intracellular compounds. A mixture of these solvents has previously been used to extract small molecules from the cultured cells.^15-19^ Two common additives that help extraction are 0.1–0.5 M of formic acid (FA) and hydrochloric acid (HCl) (Figure 2D).

The nonspecific binding with the serum proteins, the cell culture plate, the extracellular matrices, and the cell membrane can affect the determination of inhibitor intracellular concentration (Figure 1). The nonspecific binding with the serum proteins can be revealed by measurement of inhibitor intracellular concentrations with different concentrations of the serum in the cell culture medium. The nonspecific binding with the cell culture plate can be determined by incubation of the inhibitor with the culture medium and serum but without the studied cells. The nonspecific binding of the inhibitor with the cell culture plate, the extracellular matrices, and the cell membrane can be assessed by two control experiments in Figure 2C.^17^ One is the measurement of the intracellular concentration after the cells are incubated with the inhibitor at 4 °C (method A). The previous studies have shown the uptake of the inhibitors is significantly decreased as the temperature for incubation is lowered, affecting both membrane fluidity and the active uptake mechanism.^20^ At 4 °C, the uptake of inhibitor is minimal. This experiment can be used to evaluate inhibitor nonspecific binding outside and inside the cell membrane and to derive the accurate intracellular concentration. The second experiment is that the studied cells undergo trypsin–ethylenediaminetetraacetic acid (EDTA) digestion prior to compound extraction (method B). This experiment can also be used to evaluate inhibitor nonspecific binding with the extracellular proteins and matrices and the cell culture plate. The potential problem of this experiment is the overestimation of the nonspecific binding, because the inhibitor-filled cells need to be incubated in the inhibitor-free trypsin–EDTA PBS buffer at 37 °C for 2 min. Some inhibitor might leak out by the efflux mechanism.

The cellular uptake of the inhibitors can be determined after the accurate intracellular concentration is derived (Figure 1). Further, the dose- and time-dependence of inhibitor cellular stability can be obtained by this protocol. When a decrease of inhibitor intracellular concentrations is observed, the high-performance liquid chromatography (HPLC)–tandem mass spectrometry (MS/MS) studies can be performed to identify and characterize bioactive/inactive metabolites using metabolomics technologies (beyond the scope of the current protocol).

## RESULTS

### A. Compound Aqueous Solubility and Stability in the Culture Medium and Serum.

**Figure 3.**
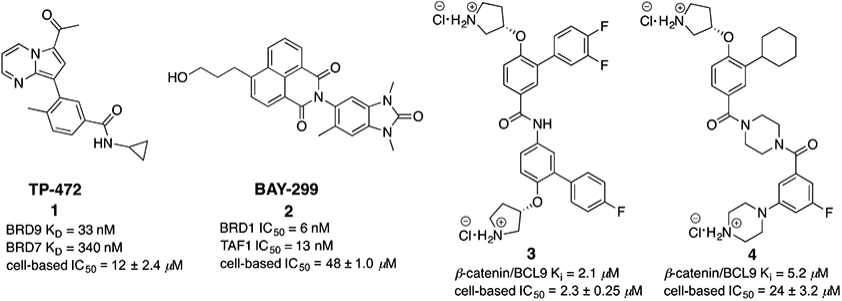
Compounds **1–4**. For **1**, the *K*_D_ values for BRD9 and BRD7 were determined by isothermal titration calorimetry (ITC) studies and reported by the Structural Genomics Consortium (SGC, www.thesgc.org/). The IC_50_ values of **2** for BRD1 and TAF1 were determined by BROMOscan and also reported by the SGC. The biochemical *K*_i_ values of **3** and **4** for the *β*-catenin/BCL9 interaction were determined by the AlphaScreen assay.^24,25^ The cell-based IC_50_ values were determined using the MTs tetrazolium assay to monitor the inhibitory effects on growth of triple negative breast cancer MDA-MB-231 cells. Each set of data is expressed as mean ± standard deviation (SD) (*n* = 3).

Compound **1** (TP-472)^21,22^ in Figure 3 is a selective inhibitor for bromodomain-containing protein 9 (BRD9) and bromodomain-containing protein 7 (BRD7). Compound **2** (BAY-299)^23^ is a selective inhibitor for bromodomain-containing protein 1 (BRD1) and transcription initiation factor TFIID subunit 1 (TAF1). Both **1** and **2** exhibit >30-fold selectivity over the other bromodomain family members and have submicromolar inhibitory activities in cell-based NanoBRET assays. The cell-based IC_50_ values of these two compounds in the MTs cell growth inhibition assay using triple negative breast cancer MDA-MB-231 cells are also shown in Figure 3. Both **3** and **4** were reported as small-molecule inhibitors for the *β*-catenin/BCL9 PPI.^24,25^ Cell-based studies indicated that **3** and **4** can suppress transactivation of canonical Wnt signaling and downregulate expression of Wnt target genes. However, compound **4** exhibits lower cell-based activities than **3** while their biochemical assay results are comparable.

**Figure 4.**
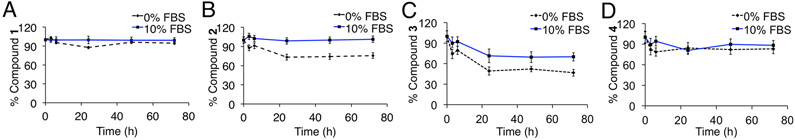
Stability of **1–4** in DMEM media over a period of 72 h with or without 10% FBS. The HPLC chromatograms at the starting time point in 5 mL of DMEM media with 10% FBS are shown in Supporting Information, Figure S1. Each set of data is expressed as mean ± standard deviation (SD) (*n* = 3).

Compounds **1–4** are soluble in water and DMEM media at concentrations up to 200 *μ*M. The stability of **1–4** in the culture medium with/without serum was evaluated. The areas under curve (AUCs) of the HPLC chromatograms for each compound in the presence and absence of 10% FBS in DMEM media were compared at the different time points. The results were reported as percent of the initial concentration (Figure 4). In DMEM media, compounds **1** and **2** were stable with 94% and 74% of the compound remaining after 72 h, respectively. When 10% FBS was added to the samples, the compounds remained in near quantitative amounts due to the binding with FBS. Compound **4** was stable over the incubation period, only decreasing to about 85% of the initial concentration at 72 h. For **4**, there was a negligible difference between the samples with and without serum. Compound **3** was less stable, with only 47% remaining after incubation for 72 h in DMEM media without FBS. When 10% FBS was added to the sample, the compound was stabilized with about 70% remaining after incubation. The nonspecific binding with FBS appeared to slow down the hydrolysis of **3** in DMEM media.

### B. Intracellular Concentrations Determined by HPLC-MS Analysis.

**Figure 5.**
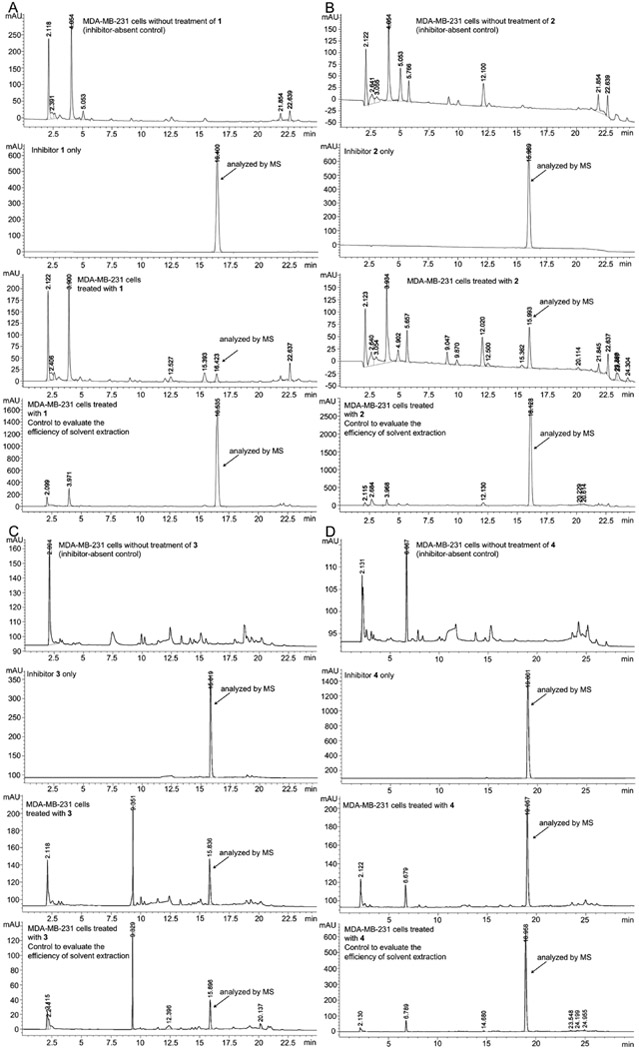
HPLC/DAD chromatograms of **1** (A) and **2** (B) and HPLC/VWD chromatograms of **3** (C) and **4** (D) in MDA-MB-231 cells under various control conditions. The retention time for **1–4** is 16.4, 15.9, 15.8, and 19.0 min, respectively. The MS data for pure and intracellular **1–4** are shown in Supporting Information, Figures S3-S6.

The HPLC/diode array detector (DAD)-based method with a vial sampler was used to quantify the intracellular concentrations of **1** and **2** in MDA-MB-231 cells. The HPLC/variable wavelength detector (VWD)-based method with the manual injection was used for **3** and **4**. For both methods to be valid, the inhibitor has to exhibit a strong absorbance in the ultraviolet–visible (UV–vis) region. The calibration curves were made for **1–4** at the wavelength where the inhibitor has strong absorption. As shown in Supporting Information, Figure S2, all calibration curves had *R*^2^ values of >0.99. The HPLC method for each inhibitor was optimized so that the retention time of the compound did not overlap with any other components in the cell lysates (Figure 5). The input concentration for each compound was chosen based on (lower than but close to) their IC_50_ values in MTs cell growth inhibition assays. Hence, the input concentrations of **1–4** were set to 10, 20, 2, and 20 *μ*M, respectively.

The control experiments were performed to evaluate the efficiency of solvents extraction. The inhibitor was added to the cells after the 37 °C incubation period and extracted with standard procedures (Figure 2B). The extraction efficiency was defined as the ratio of the amount of inhibitors extracted in the solvent extraction control experiment over the initial amount of the inhibitor added. The results indicated that MeCN/MeOH (v/v = 1:1) had the highest extraction efficiency of 1.0 for both **3** and **4**. The extraction efficiency for **1** and **2** were 0.8 and 0.9, respectively. With MeCN/MeOH (v/v = 1:1) as the extraction solvent, inhibitor intracellular concentrations were determined using the protocol shown in Figure 2A and calculated using the calibration curves and eq 3 in the Experimental Section. As shown in Table 1A, the respective intracellular moles of **1** and **2** were 0.12 ± 0.03 and 0.12 ± 0.09 nmol/million cells for the 24 h incubation in 5 mL of DMEM media with 10% FBS. The intracellular moles of **3** and **4** were 6.5 ± 0.1 and 6.9 ± 0.1 nmol/million cells for the 24 h incubation in 5 mL of DMEM media with 5% FBS, respectively. The volume of one million of MDA-MB-231 cells is about 5.5 *μ*L. The intracellular concentrations of **3** and **4** were about 600 and 65 times higher than the input concentrations of 2 and 20 *μ*M, respectively. The intracellular concentrations of **1** and **2** showed no fold increase of the compounds in the cells over the input concentrations of 10 and 20 *μ*M, respectively.

The inhibitor recovery from cells and media was determined following incubation of cells with the inhibitors by comparing the AUCs in the HPLC chromatograms. Routine mass balance calculation gave the recoveries shown in Table 1B. A quantitative or nearly quantitative amount of each inhibitor can be consistently recovered from the extra- and intracellular environments. The inhibitors have drastically different abilities for the cellular uptake. For **1**, **2**, and **4**, only 2%, <1%, and 9% of the compounds were accumulated in the cells when the input concentration was 10, 20, and 20 *μ*M, respectively. For **3**, 98% of the compound was accumulated into the cells when the input concentration was set to 2 *μ*M. For **3** and **4**, the amount that remained in the media was constant over a time period of 72 h, as shown in Figure S7. The stability of the amounts of **1** and **2** that remained in the medium over time were not evaluated.

### C. Nonspecific Binding.

The nonspecific binding with the serum proteins in the culture medium can be a significant source of error in quantifying inhibitor intracellular concentration. To determine the significance of this error for **1–4**, various concentrations of FBS were incubated with MDA-MB-231 cells and the inhibitor for 24 h. As shown in Table 1A, the negligible differences between the intracellular concentrations of **2–4** in 1%, 5%, and 10% FBS indicated that inhibitor nonspecific binding with the serum proteins in FBS did not affect the cellular uptake. The nonspecific binding of the inhibitors with the cell culture plates can be significant and sometimes can account for at least 50% of the total nonspecific binding observed.^17^ As shown in Table 1C, the nonspecific binding of **1–4** to the plates was 1%, 0.1%, 4%, and 1%, of their input concentrations of 10, 20, 2, and 20 *μ*M, respectively.

To evaluate the nonspecific binding of **1–4** with the extracellular matrices, MDA-MB-231 cells incubated with the inhibitors for 6 h were harvested using trypsin–EDTA digestion prior to extraction (the protocol is shown as method B in Figure 2C). The harvested cells are expected free of the extracellular binding with the inhibitors. The direct solvent extraction experiments were performed in parallel for comparison (the protocol is shown in Figure 2A). Through direct solvent extraction, the intracellular accumulations of **1–4** were 0.11 ± 0.04, 0.10 ± 0.07, 6.7 ± 0.1, and 9.5 ± 0.3 nmol/million cells, respectively (*n* = 3). However, using the trypsin–EDTA harvest method, the intracellular concentrations of **1–4** were 0.038 ± 0.03, 0.050 ± 0.06, 5.5 ± 0.05, and 5.9 ± 0.02 nmol/million cells, respectively (*n* = 3).

Because the cellular uptake of small molecules is minimal at 4 °C, this experiment can be used to evaluate the nonspecific binding outside and inside the cell membrane and to derive the accurate intracellular concentration (the protocol is shown as method A in Figure 2C). The incubation with MDA-MB-231 cells at 4 °C for 6 h indicated the cell-bound moles were 0.085 ± 0.04, 0.052 ± 0.01, 1.0 ± 0.01, and 0.37 ± 0.01 nmol/million cells (*n* = 3) for **1–4**, respectively. The accurate intracellular moles were derived by eq 5 and were 0.025 ± 0.04, 0.048 ± 0.06, 5.7 ± 0.1, and 9.1 ± 0.3 nmol/million cells for **1–4**, respectively, when 10 *μ*M of **1**, 20 *μ*M of **2**, 2 *μ*M of **3**, and 20 *μ*M of **4** were incubated with MDA-MB-231 cells for 6 h.

The two control experiments described in Figure 2B,C provide an opportunity to study the relationship between inhibitor structures and the nonspecific binding with the cell culture plates, the extracellular matrices, and the cell membrane. A small set of analogues was evaluated to foster this kind of studies. Compounds **5** and **6** in Table 2 are the analogues of **3** and were previously reported as the *β*-catenin/BCL9 inhibitors.^24^ Compounds **6** and **7** were reported as the inhibitors for the PPI between *β*-catenin and T-cell factor (Tcf)/lymphoid enhancer-binding factor (Lef), another downstream effector of the canonical Wnt signaling pathway.^26^ Solvent MeCN/MeOH (v/v = 1:1) can effectively extract all these compounds from water and MDA-MB-231 cells. Identical with **3** and **4**, the UV wavelengths for the HPLC analysis of **5** and **6** were set to 254 nm. The UV wavelengths for **7** and **8** HPLC analyses were set to 340 nm where they have the characteristic maximal UV absorption. The calibration curves for **5–8** are shown in Supporting Information, Figure S8. Compound **6** had more nonspecific binding with the CELLSTAR cell culture T-25 flask (material: polystyrene) than **5** and then than **3**. Compound **7** had almost no nonspecific binding with the cell culture plate, while its ethyl ester **8** exhibited more nonspecific binding. The incubation of the inhibitors with MDA-MB-231 cells at 4 °C indicated that **3** and **5** had similar nonspecific binding outside and inside the cell membrane. Compounds **6–8** exhibited little or no nonspecific binding with MDA-MB-231 cells in the 4 °C incubation experiments.

### D. Inhibitor Cell Permeability and Inhibitor Metabolic Stability in Cells.

**Figure 6.**
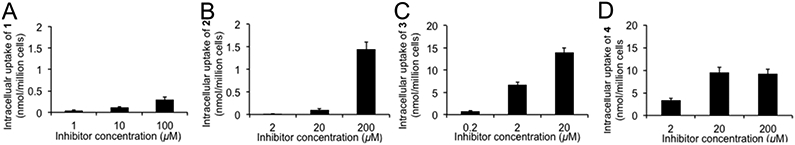
Dose dependence of the cellular uptake of **1–4**. Compounds **1** and **2** was incubated in DMEM media containing 10% FBS for 6 h. Compounds **3** and **4** were incubated in DMEM media containing 5% FBS for 6 h. Each set of data is expressed as mean ± SD (*n* = 3).

The dose dependence of inhibitor intracellular concentrations was also examined (Figure 6). Compounds **1–3** accumulated in MDA-MB-231 cells in a dose-dependent manner up to the highest concentrations examined (100, 200, and 20 *μ*M for **1–3**, respectively). For **2**, a 10-time increase in the input concentration (20–200 *μ*M) resulted in a 13-times increase in inhibitor cellular uptake. The intracellular concentration of **4** plateaued when the input concentration was increased from 20 to 200 *μ*M, suggesting that the accumulation of **4** become saturated at input concentrations above 20 *μ*M. Both **3** and **4** are the *β*-catenin/BCL9 inhibitors. They have similar functional groups but different scaffolds. The intracellular moles of **3** are 1.9 and 1.5 times greater than that of **4** at the input concentrations of 2 and 20 *μ*M, indicating that **3** is more cell permeable than **4** when the same input concentrations are compared.

**Figure 7.**
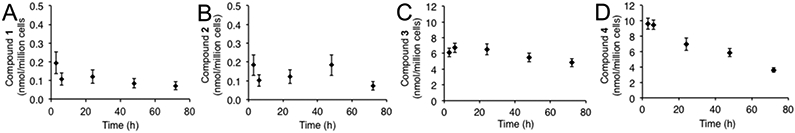
Time-dependent stability of **1–4** in MDA-MB-231 cells. The input concentrations were 10, 20, 2, and 20 *μ*M for **1–4**, respectively. Each set of data is expressed as mean ± SD (*n* = 3).

The metabolic stability of **1–4** in MDA-MB-231 cells was measured over time. The representative graphs are shown in Figure 7. Compounds **3** and **4** rapidly accumulated in cells. Maximum intracellular concentrations were achieved after 3–6 h of incubation. Compounds **1–3** exhibited good cell stability with the intracellular moles remaining relatively stable over the 72 h period. Inhibitor **4** was less stable to the cellular environment as the intracellular moles decreased at a rate of about 85.5 ± 8.2 pmol/(million cells × h).

The parallel artificial membrane permeability assay (PAMPA) was conducted to evaluate the permeability of the compounds through an artificial membrane and compare with the results from the cell-based studies. The artificial membrane was composed of 1% egg lecithin in *n*-dodecane. Each compound (200 *μ*M) was placed on the donor side of the membrane. After 5 h of incubation at room temperature, the amount of the compound was quantified through HPLC/DAD analyses. The percent transport (%*T*) and the apparent permeability coefficient (*P*_app_) were calculated using the previously published equations.^27,28^ The PAMPA results indicated that **2–4** exhibited poor permeability though the artificial membrane, while **1** had good permeability (Table 3).

## DISCUSSION

Intracellular PPIs represent a major class of targets due to their relevance to the biological processes that drive unmet biomedical needs. The determination of the cellular bioavailability is a critical step for the interpretation of cell-based data and guide the optimization of PPI inhibitors.^1-9^ The experiments that can distinguish the intracellular availability of PPI inhibitors at the site of action from the downstream response of targeting, such as the effects on reporter gene transcription, would add tremendous new insight into the chemical biology and drug discovery studies. However, the field has lack of an effective and generalizable method for determination of inhibitor cellular bioavailability. Several studies have previously been reported to determine inhibitor intracellular concentrations, but the control experiments were not offered for the key steps in the most of the studies.^15-19,29-37^ As a result, no method has been provided to determine the accurate intracellular concentration. In this study, we devised the control experiment to evaluate solvent extraction efficiency (Figure 2B) and integrated the control experiment to evaluate inhibitor nonspecific binding^17^ (Figure 2C). For the first time, this protocol allowed the determination of accurate intracellular concentrations. On the basis of the determined intracellular concentrations, the time dependence and the dose dependence of inhibitor intracellular concentrations can be derived using the workflow in Figure 1.

Gene epigenetic regulation plays an important role in normal cellular processes and contributes to a variety of human diseases, including cancer and inflammation. There are two types of epigenetic regulation: the chemical modification of DNA and the post-translational modification of histones including acetylation, methylation, and phosphorylation.^10,11^ Bromodomains read acetylated *ε*-amino group of lysines on histone tails and direct gene transcription. To date, a total of 61 bromodomain-containing proteins have been discovered in humans, and these structures share the high structural conservation, posing a significant challenge for the development of selective inhibitors.^38^ Compounds **1** and **2** are reported as the selective inhibitors for BRD9/7 and BRD1/TAF1, respectively. However, the cell-based activities of both **1** and **2** are much lower than the biochemical assay results. *β*-Catenin is a central mediator for the canonical Wnt signaling pathway. After *β*-catenin is translocated into the cell nucleus, it interacts with transcriptional factor Tcf/Lef and recruits coactivators, BCL9/BCL9-like (B9L), Pygopus (Pygo), CREB-binding protein (CBP), etc. to activate transcription of Wnt target genes. The *ε*-catenin/BCL9 PPI in the cell nucleus is a key downstream effector for the hyperactivation of the canonical Wnt signaling pathway. Recently, we have reported **3** and **4** in Figure 1 as the new inhibitors for the *β*-catenin/BCL9 PPI.^24,25^ These two inhibitors exhibit similar *K*_i_ values in biochemical AlphaScreen assays but different cell-based activities. The difference between the cell-based and biochemical assay results of **1–4** intrigued us to determine their cellular bioavailability in triple negative breast cancer MDA-MB-231 cells.

Compounds **1–4** are relatively stable in DMEM media with 10% FBS. A HPLC-MS method in Figure 2A and the control experiment in Figure 2B were used to identify suitable solvent combinations for compound extraction and measure inhibitor intracellular concentrations. MeCN/MeOH (v/v = 1/1) was found effective to extract these inhibitors from MDA-MB-231 cells. The HPLC traces in Figure 5 indicated that all compounds can clearly be separated from the cellular components, and their intracellular concentrations can be quantified. These results were reproducible in the repeated experiments.

The nonspecific binding of small-molecule inhibitors with the serum proteins in DMEM media, the cell plate, the extracellular matrices, and the cell membrane can increase the level of inhibitor extracted from the cell samples and result in an overestimation of the intracellular concentration. The intracellular concentrations of **2–4** were not significantly affected by the nonspecific binding with the serum proteins, while the intracellular concentration of **1** was almost doubled when the concentration of FBS was increased to 10% (Table 1A). Compounds **1–4** exhibited very low nonspecific binding with the cell culture plate (1%, 0.1%, 4%, and 1% for **1–4**) at the input concentrations for cell-based evaluations (10, 20, 2, and 20 *μ*M for **1–4**). The nonspecific binding of the inhibitors in the cell culture studies was evaluated by two control experiments, the 4 °C incubation and the trypsin–EDTA digestion in Figure 2C. The results for **1–3** were similar in two experiments (the decreases of intracellular concentrations of **1–3** were 77%, 52%, and 15% in the 4 °C incubation experiments and 65%, 50%, and 18% in the trypsin–EDTA digestion experiment, respectively, when the data was compared with the corresponding results from the experiments in Figure 2A). On the other hand, the trypsin–EDTA digestion experiments indicated that the intracellular concentration of **4** decreased 38%, while the incubation experiments at 4 °C offered only 4% decreases. The more significant decrease of the intracellular concentration of **4** in the trypsin–EDTA digestion experiments could be caused by the efflux of the compound from the cells because the inhibitor-filled cells were incubated with the inhibitor-free trypsin–EDTA PBS buffer at 37 °C. Further, in the trypsin–EDTA digestion experiment the cells need to be washed with cold PBS buffer and centrifuged three times. The experimental operation in this step could be challenging for some cells. Therefore, the 4 °C incubation experiment is in general more preferred in determination of inhibitor accurate intracellular concentrations.

The determination of the accurate intracellular concentration allowed evaluation of inhibitor stability kinetics in cells. Figure 6 shows the dose dependence of inhibitor uptake. Compounds **1** and **2** were accumulated by the first order, while that of **3** in a logarithmic pattern (saturation kinetics) up to the highest concentrations examined. The uptake of **4** became saturated at input concentrations >20 *μ*M. Figure 7 shows the time dependence of inhibitor uptake. Compounds **1–3** exhibited good cell stability, with the intracellular moles remaining relatively constant over the 72 h period. Inhibitor **4** was less stable as the intracellular moles decreased by the first order at a rate of about 85.5 ± 8.2 pmol/(million cells × h).

Two control experiments in Figure 2B,C also make it possible to study chemical binding of small-molecule PPI inhibitors with the plasticware, the extracellular matrices, and the nonspecific binding with the cell membrane and to derive the rules of thumb for inhibitor structure–nonspecific binding relationship. To date, only one paper has been reported to study the nonspecific binding of peptides with plasticware and glassware using ^125^I labeling.^39^ Because of the complexity of the studied peptides, no conclusion was drawn for which physicochemical properties play more important roles in the nonspecific binding. The in-parallel study on **3** (log *D*_pH=7.0_ = 1.21), **5** (log *D*_pH=7.0_ = 1.70), and **6** (log *D*_pH=7.0_ = 7.17) indicated that the hydrophobic interaction caused more nonspecific binding with the cell culture plate (CELLSTAR T-25 flask; and material, polystyrene), as shown in Table 2. The calculated physicochemical properties of **1–8** are shown in Supporting Information, Table S1. The same result was observed for **7** (log *D*_pH=7.0_ = 0.34) and **8** (log *D*_pH=7.0_ = 3.96). Compounds **7** and **8** have a large *π* ring, and it seems that arene–arene stacking interaction is less important for this nonspecific interaction. The positively charged compound **3** resulted in a higher nonspecific binding (15%) with the extracellular matrices and the cell membrane while the negatively charged **7** had the opposite effect. This result can be rationalized by the electrostatic attractive interactions between **3** and the negatively charged phospholipid bilayer in the cell membrane. Compound **7** has the repulsive interactions with the phospholipid bilayer. It is interesting that **5** exhibited higher nonspecific interactions with the extracellular matrices and the cell membrane than its hydrophobic analogue, **6**. It was noticeable that **5** has more H-bond donors and acceptors and more rotatable bonds (Supporting Information, Table S1). However, the underlying reason for this observation needs further studies.

**Figure 8.**
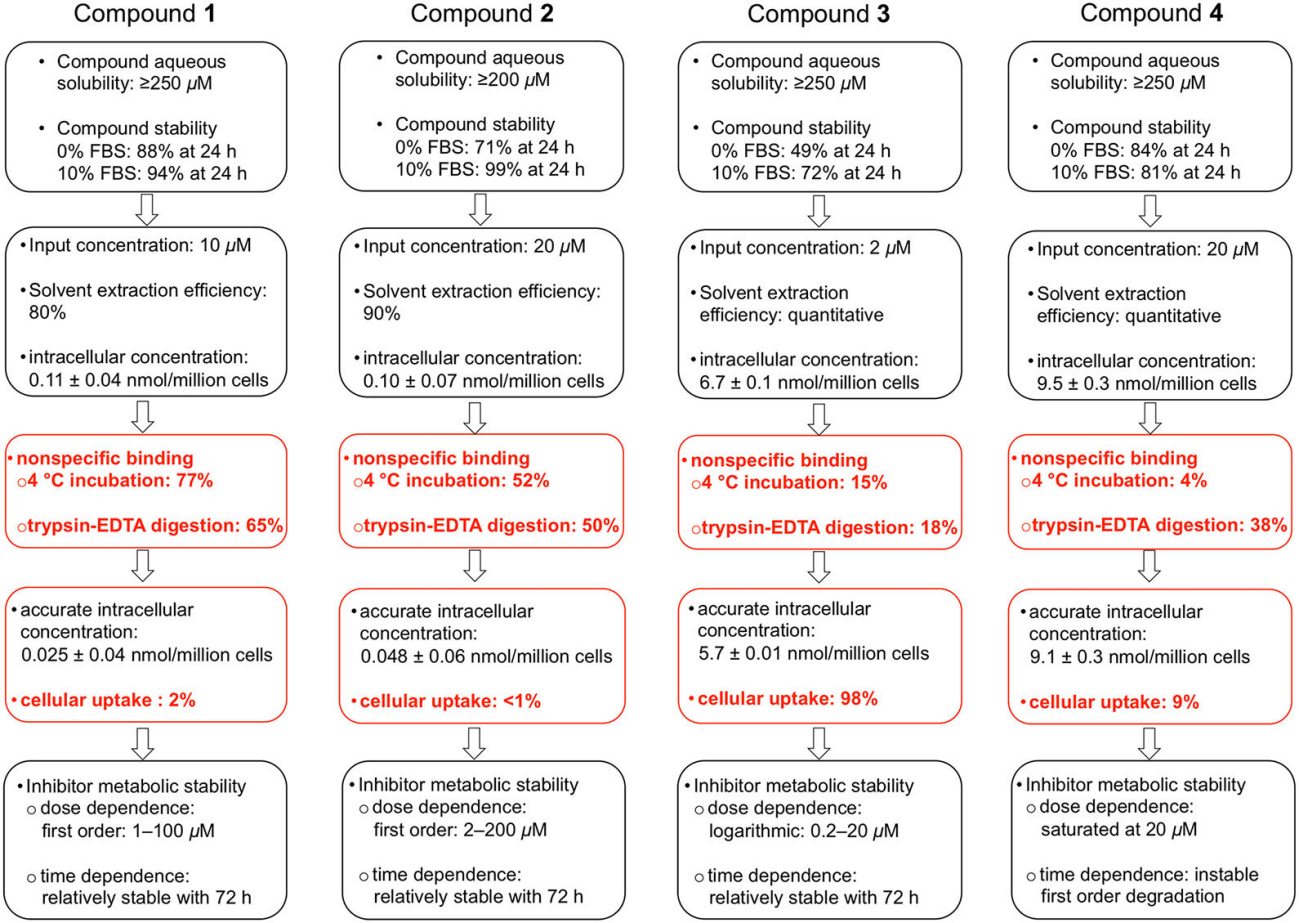
Cellular bioavailability data for **1–4**.

The IC_50_ values of **1** and **2** in MTs cell growth inhibition assays were much higher than their biochemical assay results. The recovery experiments in Table 1B indicated that only 2% and <1% of **1** and **2** were taken up by the cells. Both the 4 °C incubation and the trypsin–EDTA digestion experiments indicated that more than 50% inhibitors that had been taken up by cells bound with the extracellular matrices and the cell membrane in a nonspecific manner (Figure 8). In addition to the disease origin of the cell line, these differences could also explain why the cell-based activities of **1** and **2** are low in triple negative breast cancer MDA-MB-231 cells.

Small-molecule inhibitors need to go through two steps before expressing the pharmacological effect, one is the exposure at the target site of action (the cellular bioavailability), and the second is binding to the pharmacological target (the target engagement).^3,4^ The target engagement effects can be assessed by the cellular thermal shift assay (CETSA).^40^ Both **3** and **4** are the *β*-catenin/BCL9 inhibitors. Inhibitors **3** is 1.7-times more cell permeable than **4** when equal input concentrations were compared (Figure 6). More than 90% of **3** was accumulated into the cells, while only about 9% for **4**. The differences of **3** and **4** in the cellular permeability and uptake could account for the difference of the biochemical AlphaScreen and cell-based assay results if they display similar target engagement effects. Further, the higher cytotoxic selectivity of **4** (>10-fold) than **3** (2–4-fold) for Wnt signaling-activated cancer cells over Wnt signaling-latent cancer cells could also be associated with the difference of their cellular uptake.^24,25^

The PAMPA assay is a high throughput and low cost alternative for in vitro assessment of compound cell permeability. The results in Table 3 showed that **1** had 32.3% transport and the other three compounds exhibited very poor permeability through the artificial membrane. These PAMPA assay results do not reflect the intracellular concentration results determined by the protocol in Figure 2, and the cell-based MTs assay results in Figure 3. The difference was likely because the PAMPA assay only mimicked the passive transcellular route of compound transport. Therefore, it is important to evaluate inhibitor cellular bioavailability for the better understanding of cell-based data.

## CONCLUSIONS

We have reported a robust and generalizable HPLC-MS protocol to determine inhibitor cellular bioavailability. To the best of our knowledge, this is the first such convenient protocol that has been reported. Two control experiments were integrated with the quantification of inhibitor intracellular concentrations to study the extraction efficiency of the applied solvents and to remove noise from signal. On the basis of the derived accurate intracellular concentrations, the time dependence and the concentration dependence of the inhibitors can be derived. The techniques described in the protocol provide a method to study the relationship between inhibitor functional groups/substructures and the nonspecific binding with the plasticware, the extracellular matrices, and the cell membrane. This work has a potential to disclose how inhibitor functional groups/substructures impact the nonspecific binding in cell-based studies. The knowledge gained from these studies will enhance our understanding and interpretation of cell-based chemical biology data and facilitate the design and synthesis of bioavailable small-molecule inhibitors. The protocol will be generally useful for those medicinal chemistry programs that rely on in vitro cell-based assays and for the chemical biology programs to interpret the data in the context of cultured cells. The HPLC-MS method coupled with the use of a vial sampler allowed quantification of the intracellular concentrations for the compounds with low cellular levels, such as **1** and **2**. This protocol can also be readily adapted to the more sensitive HPLC-MS/MS techniques when needed.

## EXPERIMENTAL SECTION

### Determination of the Stability of the PPI Inhibitors in Media and Serum.

Compounds **1–4** were added in the CELLSTAR cell culture T-25 flask (Greiner Bio-One, catalogue no. 690160) in 5 mL of Dulbecco’s Modified Eagle’s Medium (DMEM, Sigma-Aldrich, catalogue no. D5523) with or without 10% FBS. The final concentrations of **1–4** were 100 *μ*M. The final concentration of dimethyl sulfoxide (DMSO) was set to 0.02% (v/v). The T-25 flasks were incubated at 37 °C in a CO_2_ incubator for the specified amount of time (3–72 h). At the specified time point, 100 *μ*L of solution for each sample was drawn for the HPLC analysis. All of the experiments were performed in triplicate. The HPLC traces at the starting time point are shown in Supporting Information, Figure S1.

### Determination of the Calibration Curves and the Limits of Detection and Quantitation.

Calibration curves were prepared for **1–8** (Supporting Information, Figures S2 and S8). Initial solutions of the pure compounds were serially diluted at least five times (for **1** from 3.07 mM, **2** from 2.15 mM, **3** from 1.72 mM, **4** from 1.27 mM, **5** from 1.91 mM, **6** from 2.29 mM, **7** from 3.14 mM, and **8** from 3.04 mM). The AUCs of the HPLC analyses were associated with the concentrations of **1–8**. Two to three replicates were performed at each concentration. The results are shown in Supporting Information, Figure S2.

The limit of detection (LOD) and the limit of quantitation (LOQ) for each inhibitor were calculated with eqs 1 and 2 below.

(1) $$\text{LOD}=3.3\times \frac{s(y)}{\text{slope}}$$

(2) $$\text{LOQ}=10\times \frac{s(y)}{\text{slope}}$$

The slope and the standard error for the *y* estimate (*s*(*y*)) for the calibration curve of each inhibitor were calculated using the LINEST function in Microsoft Excel.

### Cell Culture.

The MDA–MB–231 cell line was purchased from ATCC in October 2014. The cultured cell lines are authenticated by profiling polymorphic short tandem repeat (STR) sequences using Promega GenePrint 10 system every three months. MDA-MB-231 cells with a density of 0.7 × 10^6^ were seeded into a T-25 flask. DMEM (5 mL) with 10% FBS was added to the flask, and the cells were cultured until approximately 70% confluency was achieved (cell culture stage 1). The culture media were removed and replaced with 5 mL of fresh media (DMEM and 10% FBS for **1** and **2**, and 5% FBS for **3** and **4**, respectively) along with the specified inhibitor concentration (cell culture stage 2). The final concentration of DMSO was 0.02%. The inhibitor-absent control with 0.02% DMSO and 5% or 10% FBS in DMEM media but without the inhibitor was prepared in parallel. The T-25 flasks were further incubated at 37 °C for the specified amount of time (3–72 h).

Following the incubation, the media were removed and the T-25 flasks were quickly washed with cold phosphate-buffer saline (PBS) (3 mL × 3). The media and the PBS solutions were decanted into a glass tube, evaporated to dryness, and stored at −20 °C for the inhibitor recovery experiments. To the T-25 flasks 1.5 mL of cold MeCN/MeOH (v/v = 1:1) was added to denature cellular proteins and to extract the inhibitor. The extraction was allowed to progress to completion by storing the samples overnight at 4 °C. The samples were mixed thoroughly and transferred to 1.5 mL centrifuge tubes. Centrifugation at 12500*g* was performed for 15 min at 4 °C to precipitate cellular debris and membrane proteins. The supernatant was decanted into glass vials and evaporated to dryness. The samples were stored at −20 °C and later diluted with 150 *μ*L of deionized (DI) water for **1** and **2**, 200 *μ*L of DI water for **3** and **4**, or MeOH:MeCN (v/v = 1:1) for **5–8** for HPLC analysis. The dried residuals from the media and the PBS solutions for the inhibitor recovery experiments were diluted with 400 *μ*L of DI water for **1** and **2** or 600 *μ*L of DI water for **3** and **4** for HPLC analyses.

### HPLC-MS Analysis.

An Agilent 1260 Infinity II HPLC system equipped with a quaternary pump, a vial sampler, and a DAD detector was used for the quantitative analysis of **1** and **2**. The samples (20 *μ*L) were injected into a Kromasil 300-5-C18 column (4.6 mm × 250 mm). The DAD detector was set to 254 and 355 nm for **1** and 235 and 355 nm for **2**. The mobile phase consisted of a mixture of H_2_O (0.1% trifluoroacetic acid, TFA) and MeCN. Gradient elution was applied to each compound: 100% H_2_O (0.1% TFA) to H_2_O (0.1% TFA):MeCN = 50:50 from 0 to 18 min, H_2_O (0.1% TFA):MeCN = 50:50 to 100% MeCN from 18 to 20 min, and 100% MeCN from 20 to 25 min. The flow rate was 1.5 mL/min. The column was equilibrated to each starting mobile phase for approximately 10 min between runs. Using this method, the purity of **1** and **2** was ≥95%.

MS data for **1** and **2** were recorded on an Agilent 1100 HPLC mass selective detector (MSD) instrument with an electrospray ionization (ESI) source. The samples were analyzed through a direct injection method. The mobile phase was H_2_O (0.1% FA) and MeCN (0.1% FA). Isocratic elution was applied: H_2_O (0.1% FA):MeCN (0.1% FA) = 50:50 for 4 min. The flow rate was 1.3 mL/min. The mass spectrometer was operated using an ESI source in the positive ion mode. The MS source parameters were as follows: capillary voltage, 3.0 kV; drying gas flow, 10 L/min; drying gas temperature, 350 °C; and nebulizer pressure, 20 psig. The MS data was acquired with Agilent Chem-Station B.04.03.

An Agilent 1260 HPLC system equipped with a quaternary pump, a manual injector, and a VWD detector was used for **3–8**. The samples (20 *μ*L) were injected into a Kromasil 300–5–C18 column (4.6 mm × 250 mm). The VWD detector was set to 254 nm for **3–6** and 340 nm for **7** and **8**. The mobile phase consisted of a mixture of H_2_O (0.1%, TFA) and either MeOH or MeCN. Gradient elution was applied to each compound: **3**, **5**, and **6** 100% H_2_O (0.1% TFA, v/v) for 6 min, 100% H_2_O (0.1% TFA) to H_2_O (0.1% TFA):MeCN = 50:50 from 6 to 15 min, H_2_O (0.1% TFA):MeCN = 50:50 to 100% MeCN from 15 to 18 min, and 100% MeCN from 18 to 25 min; **4**, 100% H_2_O (0.1% TFA) for 3 min, 100% H_2_O (0.1% TFA) to H_2_O (0.1% TFA):MeOH = 30:70 from 3 to 20 min, H_2_O (0.1% TFA):MeOH = 30:70 to 100% MeOH from 20 to 23 min, and 100% MeOH from 23 to 30 min; **7** and **8**, 100% H_2_O (0.1% TFA) to H_2_O (0.1% TFA):MeCN = 50:50 from 0 to 15 min, H_2_O (0.1% TFA):MeCN = 50:50 to 100% MeCN from 15 to 18 min, and 100% MeCN from 18 to 25 min. The flow rate was 1.5 mL/min. The column was equilibrated to each starting mobile phase for approximately 10 min between runs. Using this method, the purity of compound **3–8** was ≥95%.

MS data for **3–8** were recorded on a Waters Acquity tandem quadrupole mass detector (TQD) instrument with an ESI source. The samples were injected into a Waters Acquity UPLC BEH C18 column (1.7 *μ*M, 2.1 mm × 50 mm). The mobile phase was H_2_O (0.1%, FA) and MeCN (0.1% FA). Gradient elution was applied: 100% H_2_O (0.1% FA) for 0.85 min, 100% H_2_O (0.1% FA) to H_2_O (0.1% FA):MeCN (0.1% FA) = 50:50 from 0.85 to 2.34 min, H_2_O (0.1% FA):MeCN (0.1% FA) = 50:50 to 100% MeCN (0.1% FA) from 2.34 to 2.81 min, 100% MeCN (0.1% FA) from 2.81 to 3.91 min, 100% MeCN (0.1% FA) to 100% H_2_O (0.1% FA) from 3.91 to 4.38 min, 100% H_2_O (0.1% FA) from 4.38 to 5 min. The flow rate was 0.4 mL/min. The mass spectrometer was operated using an ESI source in the positive ion mode. The MS source parameters were as follows: capillary voltage, 2.50 kV; cone voltage, 30 V; source temperature, 110 °C; and cone gas flow, 10 L/h. The MS data was acquired with MassLynx v4.1 software.

### Determination of Inhibitor Intracellular Concentrations.

The calibration curves as described in Supporting Information, Figures S2 and S8, were made to determine the concentrations of the examined inhibitors ([compound]_calibration_). A correction for dilution was further made for the intracellular concentration of each compound using eq 3. The solvent extraction efficiency will be derived by the experiment shown below. The experiments were performed in triplicate. The results were expressed as mean ± SD.

(3) $${\text{compound}}_{\text{intracellular}}=\frac{[\text{compound}{]}_{\text{calibration}}\times \frac{\text{total volume of sample for HPLC analysis}}{\text{number of cells}}}{\text{solvent extraction efficiency}}$$

Routine mass balance was calculated to determine the recovery of the inhibitors from the assays. The moles of the inhibitor obtained from both cell and medium samples after incubation were compared with the initial moles of the inhibitor that were added to the culture media.

### Control Experiments to Evaluate the Efficiency of Solvent Extraction.

After 70% confluency was achieved in the first-stage cell culturing, 0.02% DMSO and 5% FBS in DMEM media without the inhibitor was added to the T-25 flasks containing MDA-MB-231 cells. The media were removed after the second-stage cell culturing, and the T-25 flasks were quickly washed with cold PBS buffer (3 mL × 3). The inhibitor was added to 1.5 mL of the extraction solvent that was then applied to the cells. The subsequent extraction, centrifugation, and HPLC steps were identical with those used to determine inhibitor intracellular concentrations. The solvent extraction efficiency was calculated by eq 4 below.

(4) $$\text{solvent extraction efficiency}=\frac{\text{extracted inhibitor moles from HPLC analysis}}{\text{input inhibitor moles}}$$

### Determination of the Nonspecific Binding and the Accurate Intracellular Concentrations.

Cell-absent control experiments were performed to determine the nonspecific binding of the inhibitors with the cell culture plates. No MDA-MD-231 cells were added, and all of the other steps were identical with those used to determine inhibitor intracellular concentrations. The percent of nonspecific binding was calculated by (the inhibitor moles bound to the cell culture plates) ÷ (the input inhibitor moles × solvent extraction efficiency) × 100%.

Trypsin–EDTA digestion experiments were performed to determine inhibitor nonspecific binding with the extracellular proteins and matrices and with the cell culture plate. After incubating with the inhibitor in the second-stage cell culturing, MDA-MD-231 cells were quickly washed three times with ice-cold PBS buffer. The cells were then removed from the plate by incubation with 0.25% trypsin and 1 mM EDTA in PBS buffer for 2 min at 37 °C. The cells were resuspended in cold DMEM media and washed with ice-cold PBS buffer once before the addition of cold MeCN/MeOH (v/v = 1/1) to extract the compound. The experiments were performed in triplicate. The results were expressed as (mean ± SD)/(solvent extraction efficiency).

Incubation experiments at 4 °C were performed to determine inhibitor nonspecific binding with the cell membrane and the cell culture plate. After 70% confluency was achieved in the first-stage cell culturing, to the T-25 flasks containing MDA-MD-231 cells was added 0.02% DMSO, 10% FBS for **1** and **2** or 5% FBS for **3** and **4**, and the inhibitor in 5 mL cold DMEM media. The T-25 flasks were incubated at 4 °C for 6 h in the second-stage cell culturing. The subsequent inhibitor extraction, centrifugation, and HPLC steps were identical with those used to determine inhibitor intracellular concentrations. The experiments were performed in triplicate. The results were expressed as (mean ± SD)/(solvent extraction efficiency). The accurate intracellular concentration of the inhibitor can be derived by eq 5.

(5) $${\text{compound}}_{\text{intracellular,accurate}}\;\;={\text{compound}}_{\text{intracellular},37^{{}_{{}^{\circ}}}C}-{\text{compound}}_{\text{intracellular},4^{{}_{{}^{\circ}}}C}$$

### Artificial Membrane Permeability Assay.

A 96-well filtration plate (EMD Millipore, catalogue no. MAIPNTR10) was used as the artificial membrane support and the receiver plate. The filter material in each well of the filtration plate was wetted with 5 *μ*L of the artificial membrane solution, which consisted of 1% egg lecithin (Sigma-Aldrich, catalogue no. P3556) in *n*-dodecane (Fisher Chemical, catalogue no. O2666-500). The filtration plate was then securely placed on top of a donor plate (EMD Millipore, catalogue no., MATRNPS50), which was prefilled with the donor solution (200 *μ*M compound solution, 280 *μ*L) in phosphate buffer (pH 7.4) in each well. Equal volumes of the blank receiving solution (phosphate buffer, pH = 7.4) were quickly added to the wells of the filtration plate. The stacked donor–receiver plates were incubated at room temperature for 5 h with gentle circular shaking. After incubation, the receiving solution was assayed against the concentrations of the initial donor solution using HPLC.

The results of the artificial membrane permeability were expressed as the percent transport (%*T*) using eq 6.

(6) $$\%T=100\times \frac{A_{R}\cdot V_{R}}{A_{\text{D0}}\cdot V_{D}}$$

where *A*_D0_ and *A*_R_ are the HPLC peak areas of the initial donor solution and the receiving solution after incubation and *V*_R_ and *V*_D_ are the volumes of the receiving and donor solution, respectively.

The %*T* is related to the apparent permeability coefficient *P*_app_ using eq 7.

(7) $$P_{\text{app}}=\frac{V_{D}\cdot V_{R}}{(V_{D}+V_{R})S\cdot t}\text{ln}\left[\frac{100\cdot V_{D}}{100\cdot V_{D}-\%T(V_{D}+V_{R})}\right]$$

where *S* is the surface area of the artificial membrane, and *t* is the incubation time.

## Supplementary Material

Supplemental Figures & Table

Molecular formula strings

## Figures and Tables

**Table 1.** (A) Intracellular Moles of 1–4 for the 24 h Incubation in 5 mL of DMEM Media; (B) Results of the Recovery Experiments for the Compounds; (C) Nonspecific Binding of the Compounds with the Cell Culture Plate

A		
compd^a^	FBS (%) in 5 mL of DMEM	intracellular uptake (nmol/million cells ± SD)^b^
**1**	1	0.069 ± 0.03
	5	0.074 ± 0.03
	10	0.12 ± 0.03
**2**	1	0.15 ± 0.08
	5	0.18 ± 0.08
	10	0.12 ± 0.09
**3**	1	6.1 ± 0.1
	5	6.5 ± 0.1
	10	5.7 ± 0.1
**4**	1	8.7 ± 0.2
	5	6.9 ± 0.1
	10	8.2 ± 0.2

B				
		final amount (*μ*mol)^b^		
compd^c^	initial amount (*μ*mol)	extracellular	intracellular	%recovery ± SD^b^
**1**	0.065	0.059 ± 0.000007	0.0012 ± 0.00001	93.0 ± 5.3
**2**	0.065	0.059 ± 0.00009	0.0004 ± 0.00028	91.2 ± 3.4
**3**	0.0103	nd^d^	0.0101 ± 0.000483	97.9 ± 4.7
**4**	0.115	0.107 ± 0.0119	0.0100 ± 0.00206	101.3 ± 9.8

C		
compd	input concentration (*μ*M)	extracted concentration (*μ*M)
**1**	1	0.035
	10	0.100
	100	0.665
**2**	2	0.009
	20	0.016
	200	0.719
**3**	0.2	0.022
	2	0.076
	20	0.128
**4**	0.2	0.133
	2	0.198
	20	0.222

a The input concentrations of **1–4** were 10, 20, 2, and 20 *μ*M, respectively.

b Each set of data is expressed as mean ± SD (*n* = 3).

c Compounds were incubated in 5 mL DMEM media with 10% FBS (**1** and **2**) or 5% FBS (**3** and **4**) for 24 h in MDA-MB-231 cells.

d nd, not determined.

**Table 2.** Relationship between Inhibitor Functional Groups/Substructures and the Binding with the Plasticware, the Extracellular Matrices, and the Nonspecific Binding with the Cell Membrane

	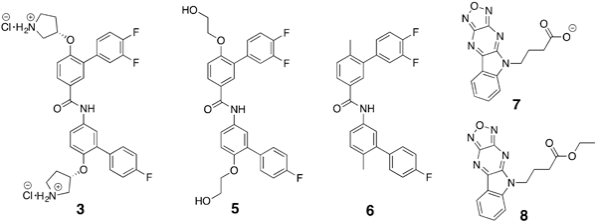			
compd	input concentration (*μ*M)^a^	solvent extraction efficiency (MeCN/MeOH, v/v = 1/1)	nonspecific binding with the culture plate (%)^b,c^	cell-bound inhibitors at 4 °C (nmol/ million cells)^c^
**3**	2	1.0	4.0 ± 0.5	1.0 ± 0.01^c^
**5**	2	1.0	8.6 ± 0.8	1.0 ± 0.01
**6**	2	0.7	16 ± 2.4	0.3 ± 0.02
**7**	2	0.9	0.49 ± 0.052	0.0 ± 0.03
**8**	2	0.8	3.9 ± 0.27	0.1 ± 0.00

a All inhibitors (2 *μ*M) were incubated in 5 mL fresh media (DMEM and 5% FBS).

b The cell culture plates are CELLSTAR cell culture T-25 flask (Greiner Bio-One, catalogue no. 690160; material: polystyrene).

c All experiments were performed in triplicate (*n* = 3).

**Table 3.** PAMPA Results of 1–4

compd	%*T* ± SD	*P*_app_ ± SD (cm·s^−1^, × 10^−6^)
**1**	32.3 ± 2	14.3 ± 1.6
**2**	0.3 ± 0.08	0.08 ± 0.02
**3**	0.2 ± 0.04	0.13 ± 0.01
**4**	0.1 ± 0.09	0.04 ± 0.02

## ABBREVIATIONS USED

AUCs: areas under curve
BCL9: B-cell lymphoma 9
BRD1: bromodomain-containing protein 1
BRD7: bromodomain-containing protein 7
BRD9: bromodomain-containing protein 9
BSA: bovine serum albumin
DAD: diode array detector
DI: deionized
DMEM: Dulbecco’s Modified Eagle’s Medium
DMSO: dimethyl sulfoxide
EDTA: ethylenediaminetetraacetic acid
ESI: electrospray ionization
FA: formic acid
FBS: fetal bovine serum
HPLC: high-performance liquid chromatography
Lef: lymphoid enhancer-binding factor
LOD: limit of detection
LOQ: limit of quantitation
MeCN: acetonitrile
MeOH: methanol
MS: mass spectrometry
PAMPA: parallel artificial membrane permeability assay
PBS: phosphate-buffer saline
PPI: protein–protein interaction
SD: standard deviation
SGC: Structural Genomics Consortium
STR: short tandem repeat
TAF1: transcription initiation factor TFIID subunit 1
Tcf: T-cell factor
TFA: trifluoroacetic acid
TQD: tandem quadrupole mass detector
UV–vis: ultraviolet–visible
VWD: variable wavelength detector
