# Supplementary material for: A Versatile Method to Determine the Cellular Bioavailability of Small-Molecule Inhibitors
Source: J Med Chem. Author manuscript; Available in PMC 2020 Dec 29. (PMC7771553; doi:10.1021/acs.jmedchem.6b00923)
Supplement: Supplemental Figures & Table [file NIHMS1652902-supplement-Supplemental_Figures___Table.pdf]

A Versatile Method to Determine the Cellular Bioavailability of Small-Molecule Inhibitors

Kevin B. Teuscher,†, § Min Zhang,† and Haitao Ji†, ‡,§,*

† Drug Discovery Department, H. Lee Moffitt Cancer Center and Research Institute, 12902 Magnolia Drive, Tampa, Florida 33612–9416, United States.
‡ Department of Oncologic Sciences, University of South Florida College of Medicine, Tampa, Florida 33612, United States.
§ Department of Chemistry, University of Utah, Salt Lake City, Utah 84112, United States.

*Correspondence to: Dr. Haitao Ji (E-mail: Haitao.Ji@moffitt.org)

Table of Contents

Supplemental Procedures.....S2
Figure S1.....S3–S4
Figure S2.....S5–S8
Figure S3.....S9
Figure S4.....S10
Figure S5.....S11
Figure S6.....S12
Figure S7.....S13
Figure S8.....S14–S17
Table S1.....S18
Supplementary Reference.....S19

Supplemental Procedure.

MTs cell viability assay. MDA−MB−231 were seeded in the 96-well plates at 4×10^3 cells/well, maintained overnight at 37 °C, and incubated with **1** (TP-472), **2** (BAY-299), and **4** at various concentrations. Cell viability was monitored after 72 h using a freshly prepared mixture of 1 part phenazine methosulfate (PMS, Sigma) solution (0.92 mg/mL) and 19 parts 3-(4,5-dimethylthiazol-2-yl)-5-(3-carboxymethoxyphenyl)-2-(4-sulfophenyl)-2H-tetrazolium (MTs, Promega) solution (2 mg/mL). Cells were incubated in 10 μL of this solution at 37 °C for 3 h, and A_{490} was measured. The effect of the compound is expressed as the concentration required to reduce A_{490} by 50% (IC_{50}) relative to DMSO-treated cells. Experiments were performed in triplicate.

The MTs cell viability assay result of **3** for triple negative breast cancer MDA-MB-231 cells have previously been reported.[24]

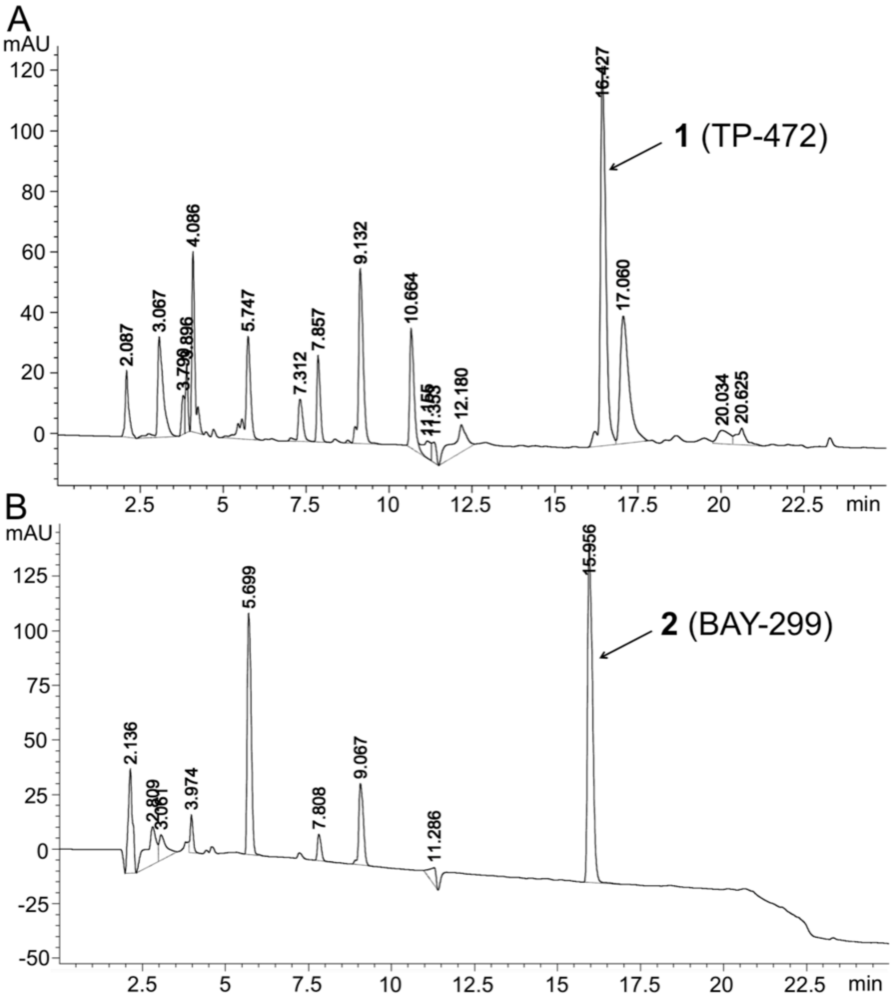
A
mAU
120
100
80
60
40
20
0
2.087
3.067
3.896
4.086
5.747
7.312
7.857
9.132
10.664
12.180
16.427
17.060
20.034
20.625
1 (TP-472)
2.5
5
7.5
10
12.5
15
17.5
20
22.5
min
B
mAU
125
100
75
50
25
0
-25
-50
2.136
2.809
3.061
3.974
5.699
7.808
9.067
11.286
15.956
2 (BAY-299)
2.5
5
7.5
10
12.5
15
17.5
20
22.5
min

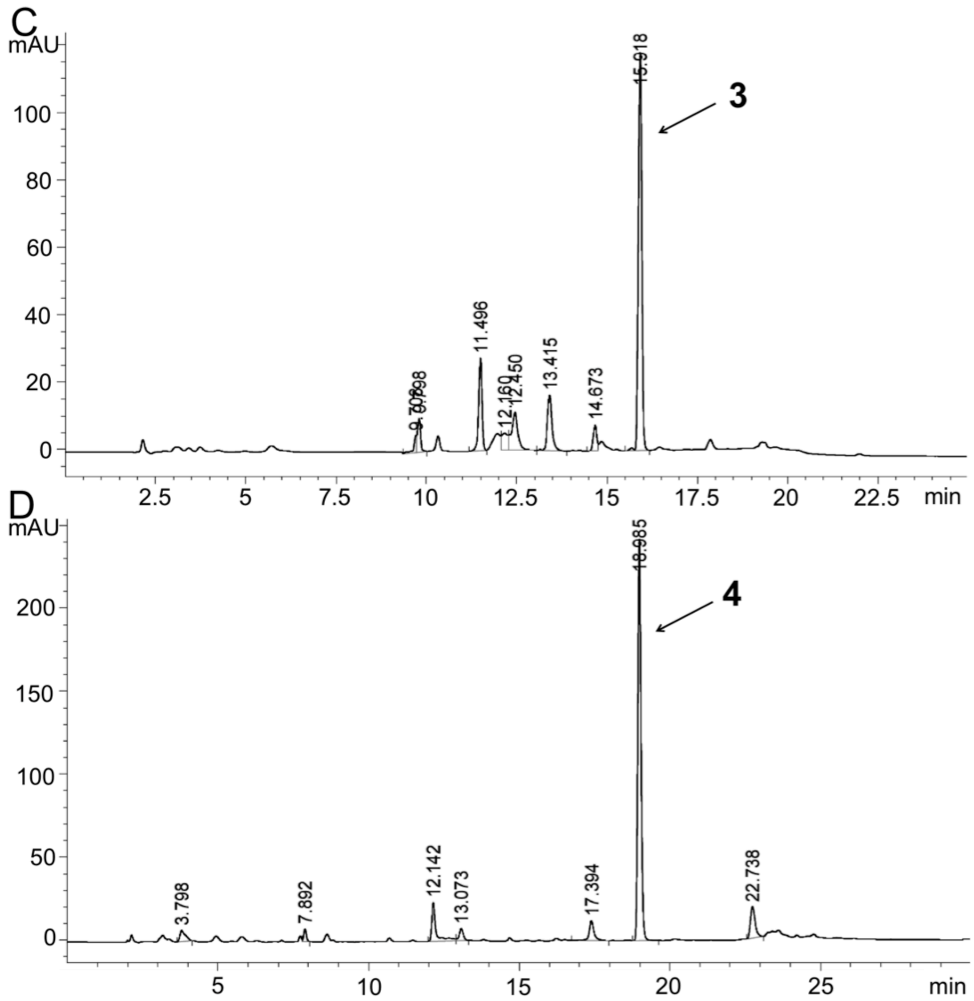

Figure S1. HPLC chromatograms of 1 (TP-472), 2 (BAY-299), **3**, and **4** in 5 mL DMEM with fetal bovine serum (FBS) (10% for **1** and **2**; 5% for **3** and **4**) at the starting time point.

A

HPLC Injection (20 μL)	Concentration of **1** (mol/L)	AUC (mAU)
1	7.67×10^{-4}	1.51×10^{4}
2		1.51×10^{4}
3		1.50×10^{4}
1	3.84×10^{-4}	7.63×10^{3}
2		7.63×10^{3}
3		7.62×10^{3}
1	1.92×10^{-4}	3.74×10^{3}
2		3.74×10^{3}
3		3.71×10^{3}
1	9.59×10^{-5}	1.84×10^{3}
2		1.84×10^{3}
3		1.84×10^{3}
1	4.79×10^{-5}	9.47×10^{2}
2		9.55×10^{2}
3		9.45×10^{2}
1	1.20×10^{-5}	2.50×10^{2}
2		2.49×10^{2}
3		2.48×10^{2}
1	3.00×10^{-6}	7.82×10^{1}
2		6.64×10^{1}
3		1.85×10^{1}

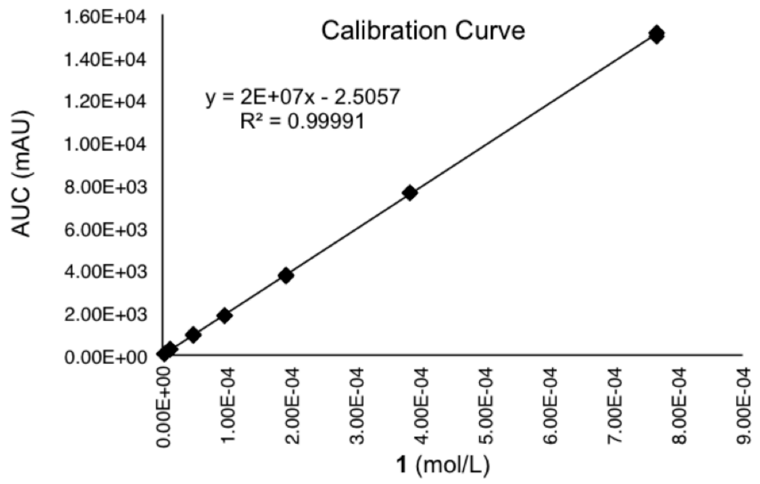

B

HPLC Injection (20 μL)	Concentration of **2** (mol/L)	AUC (mAU)
1	5.38×10^{-4}	6.21×10^{3}
2		6.21×10^{3}
3		6.13×10^{3}
1	2.69×10^{-4}	3.18×10^{3}
2		3.18×10^{3}
3		3.18×10^{3}
1	1.34×10^{-4}	1.58×10^{3}
2		1.60×10^{3}
3		1.58×10^{3}
1	6.72×10^{-5}	7.96×10^{2}
2		7.95×10^{2}
3		7.96×10^{2}
1	3.36×10^{-5}	3.71×10^{2}
2		3.68×10^{2}
3		3.71×10^{2}
1	8.40×10^{-6}	8.88×10^{1}
2		8.88×10^{1}
3		8.88×10^{1}
1	2.10×10^{-6}	2.09×10^{1}
2		2.08×10^{1}
3		2.03×10^{1}

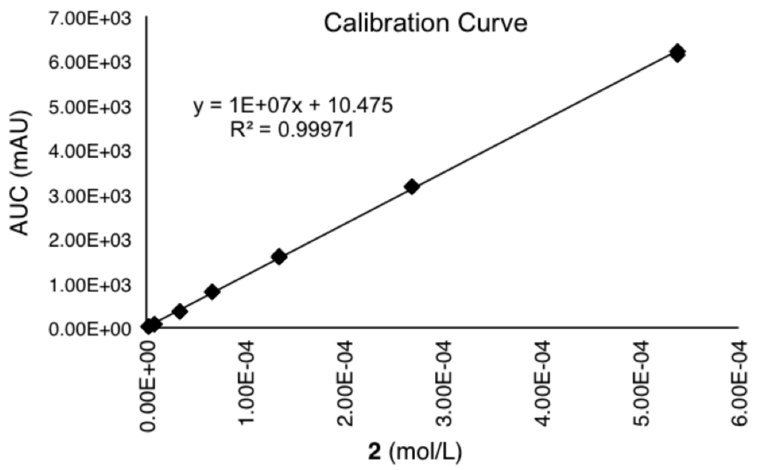

C

HPLC Injection (10 μL)	Concentration of **3** (mol/L)	AUC (mAU)
1	1.72×10^{-3}	1.60×10^{4}
2		1.70×10^{4}
3		1.50×10^{4}
1	8.62×10^{-4}	6.37×10^{3}
2		6.76×10^{3}
3		7.54×10^{3}
1	4.31×10^{-4}	2.92×10^{3}
2		2.71×10^{3}
3		2.94×10^{3}
1	2.15×10^{-4}	1.03×10^{3}
2		9.55×10^{2}
3		9.10×10^{2}
1	1.08×10^{-4}	3.15×10^{2}
2		2.25×10^{2}

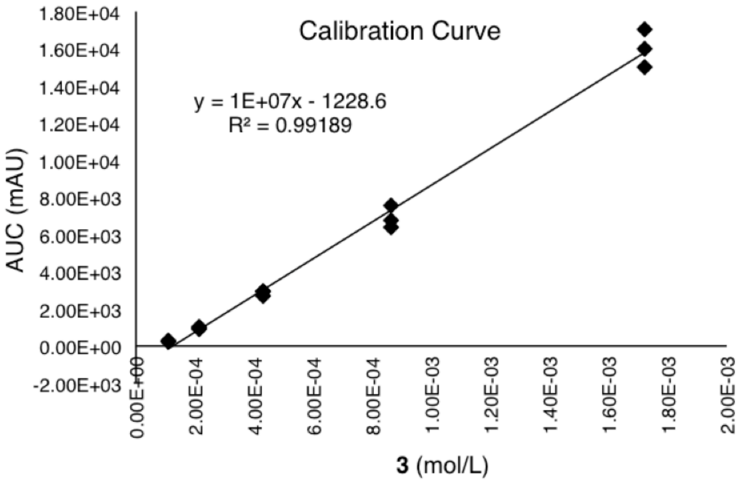

D

HPLC Injection (20 μL)	concentration of **4** (mol/L)	AUC (mAU)
1	1.27×10^{-3}	1.53×10^{4}
2		1.53×10^{4}
3		1.49×10^{4}
1	6.37×10^{-4}	7.25×10^{3}
2		7.15×10^{3}
3		7.27×10^{3}
1	3.19×10^{-4}	3.40×10^{3}
2		3.57×10^{3}
3		3.24×10^{3}
1	1.59×10^{-4}	1.55×10^{3}
2		1.54×10^{3}
3		1.61×10^{3}
1	7.97×10^{-5}	5.49×10^{2}
2		4.89×10^{2}
3		4.57×10^{2}
1	3.98×10^{-5}	1.57×10^{2}
2		1.34×10^{2}
3		1.28×10^{2}

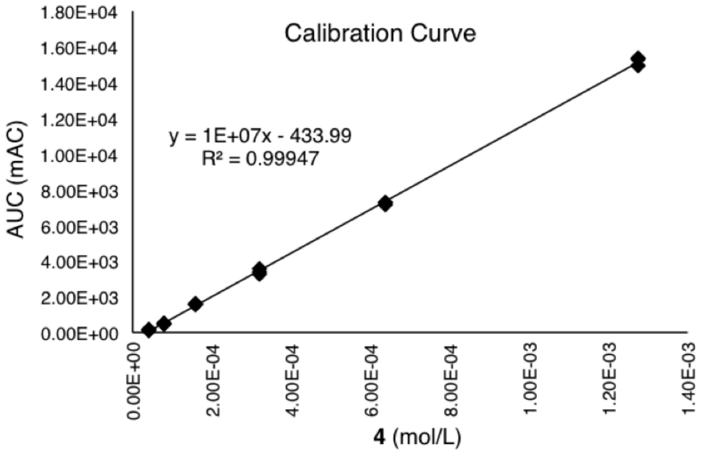

Figure S2. Determination of the calibration curves for **1–4**.

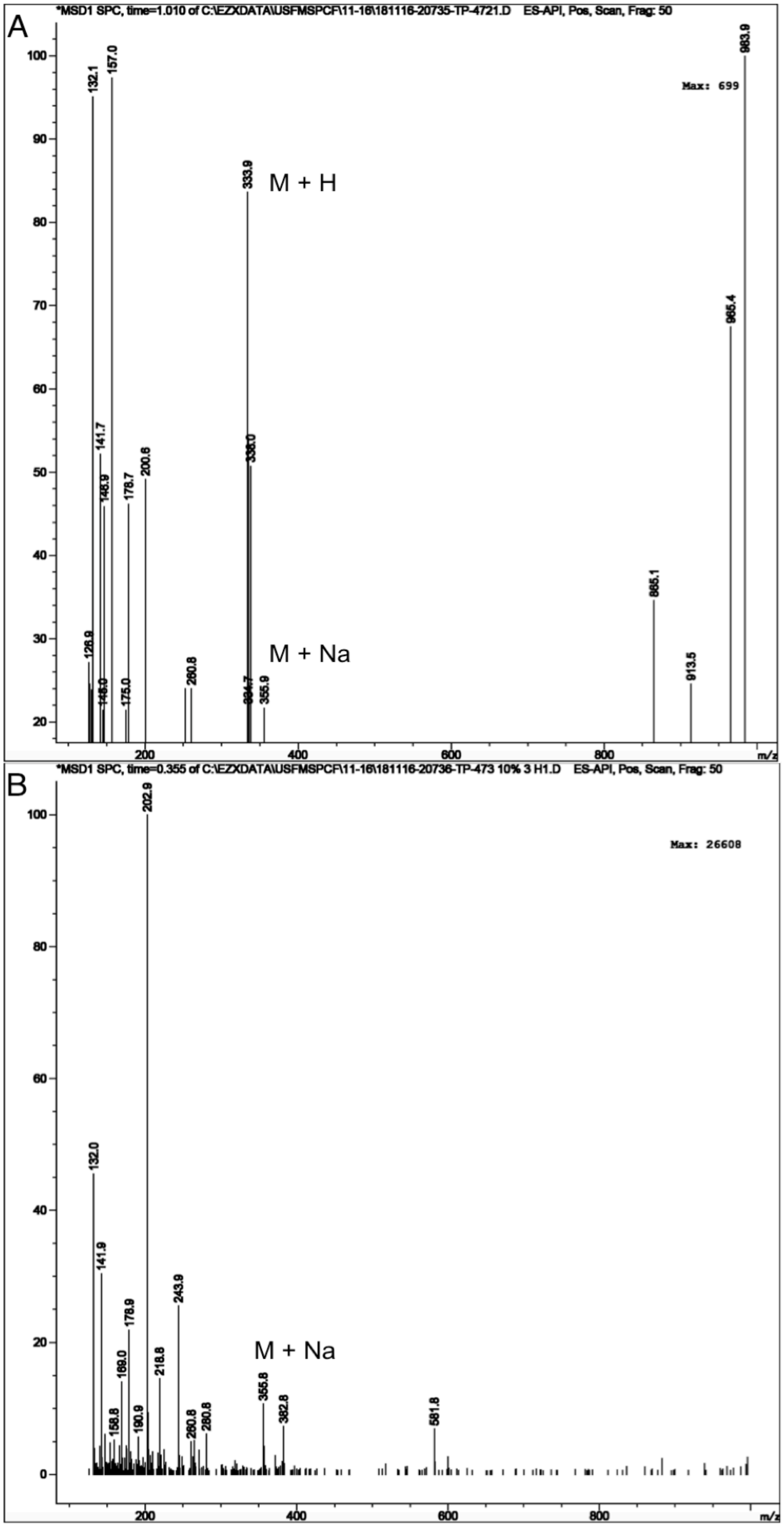

Figure S3. Mass spectrometry (MS) data for the HPLC peaks in Figure 3A. (A) pure **1**. (B) MDA-MB-231 cell samples after the treatment with **1** for 24 h.

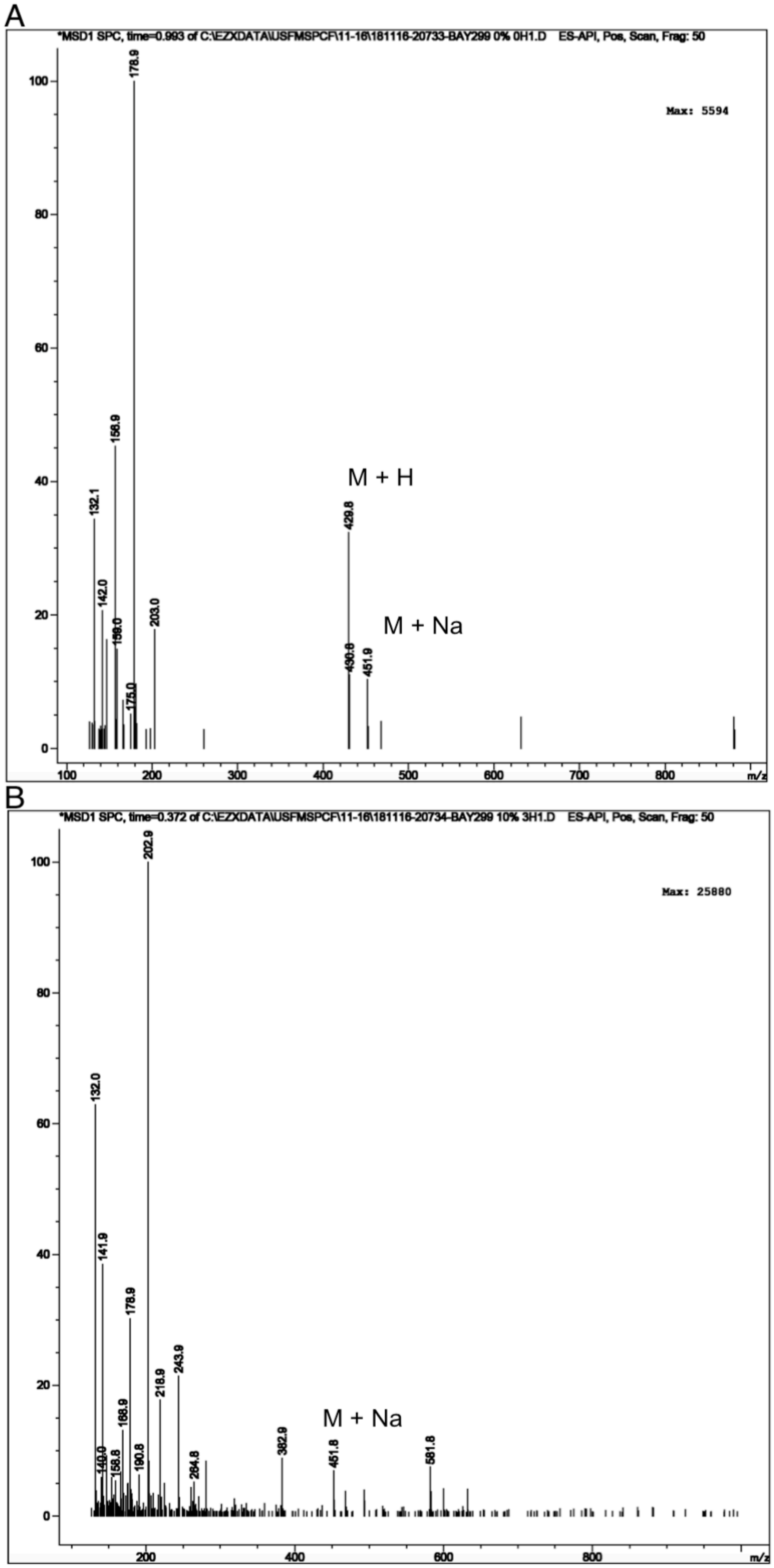

Figure S4. Mass spectrometry (MS) data for the HPLC peaks in Figure 3B. (A) pure **2**. (B) MDA-MB-231 cell samples after the treatment with **2** for 24 h.

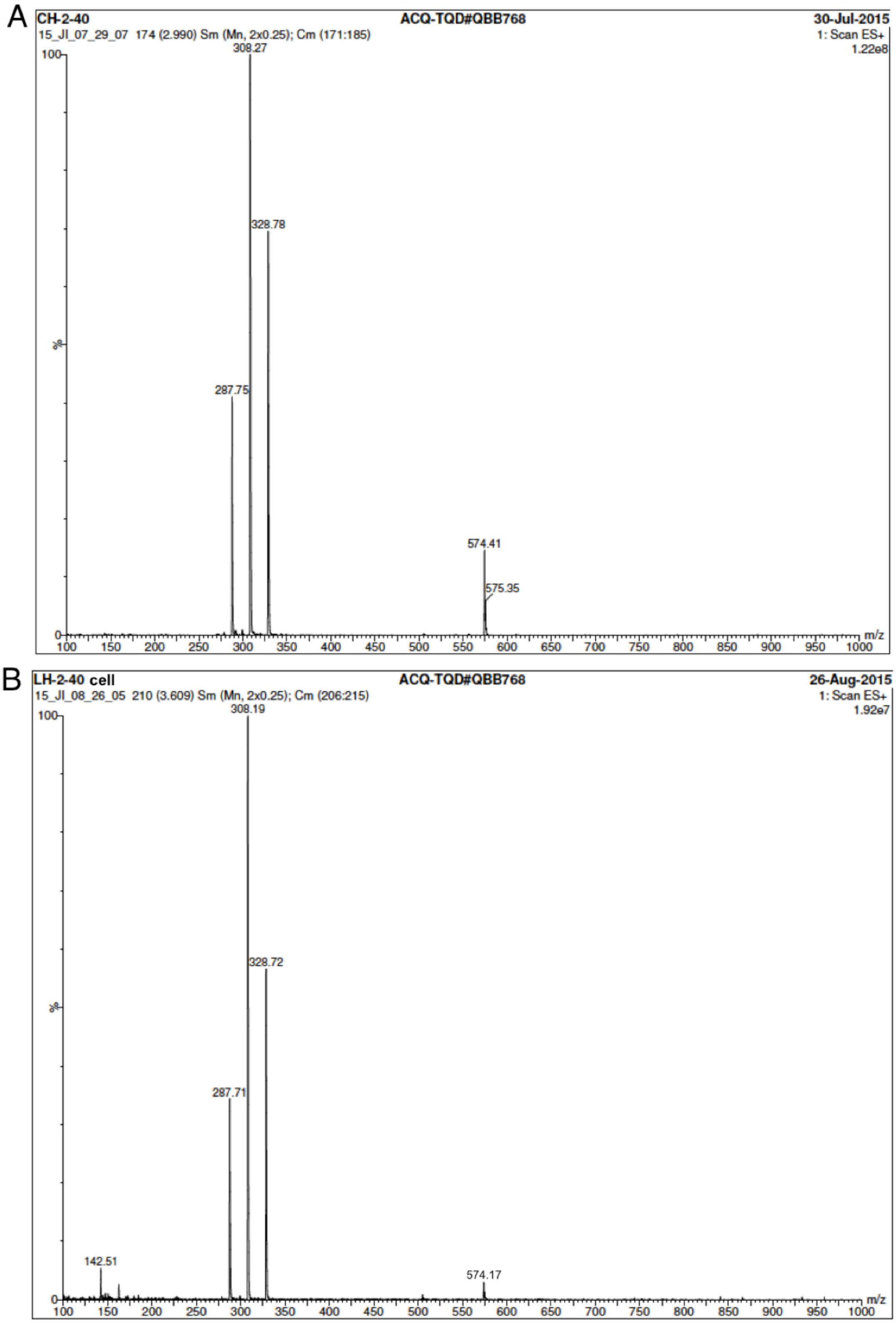

Figure S5. Mass spectrometry (MS) data for the HPLC peaks in Figure 3C. (A) pure **3**. (B) MDA-MB-231 cell samples after the treatment with **3** for 24 h.

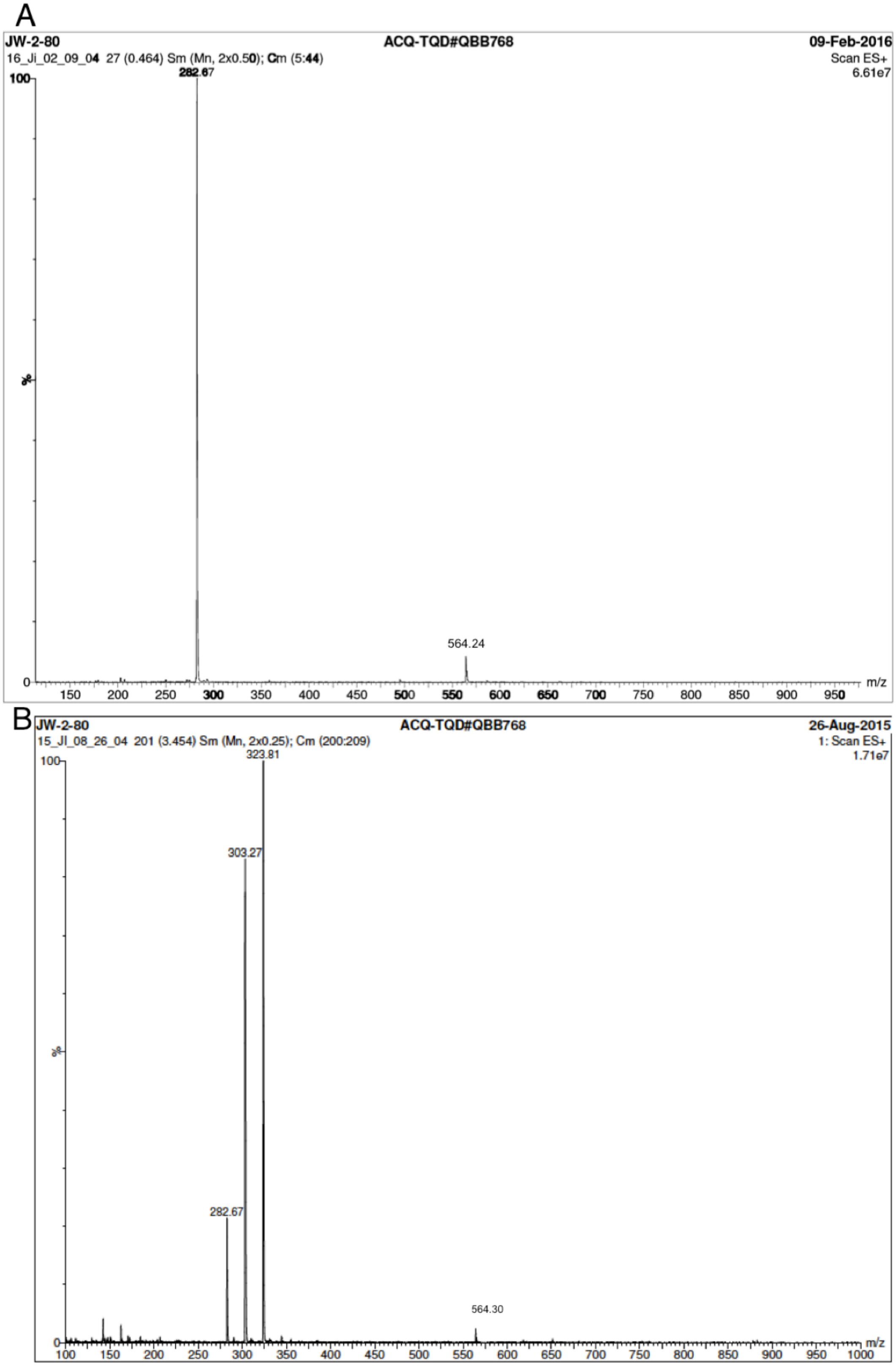

Figure S6. Mass spectrometry (MS) data for the HPLC peaks in Figure 3D. (A) pure **4**. (B) MDA-MB-231 cell samples after the treatment with **4** for 24 h.

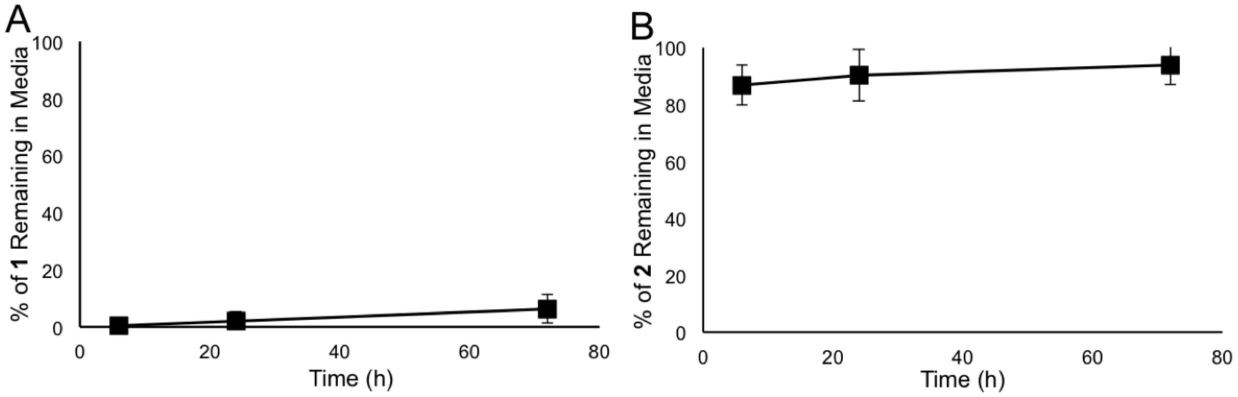

Figure S7. Time-dependence of the percent of the *β*-catenin/BCL9 inhibitors remaining the DMEM medium. Inhibitors **3** (A) and **4** (B) were incubated over a period of 72 h with the initial concentration of 2 and 20 μM. Each set of data is expressed as mean ± standard deviation (SD) (n = 3).

A

HPLC Injection (10 μL)	Concentration of **5** (mol/L)	AUC (mAU)
1	1.92×10^{-3}	3.78×10^{4}
2		3.50×10^{4}
3		3.72×10^{4}
1	9.59×10^{-4}	2.01×10^{4}
2		1.92×10^{4}
3		1.90×10^{4}
1	4.79×10^{-4}	9.82×10^{3}
2		9.52×10^{3}
3		9.69×10^{3}
1	2.40×10^{-4}	4.97×10^{3}
2		5.03×10^{3}
3		5.02×10^{3}
1	1.20×10^{-4}	2.32×10^{3}
2		2.33×10^{3}
3		2.32×10^{3}
1	5.99×10^{-5}	1.23×10^{3}
2		1.24×10^{3}
3		1.14×10^{3}

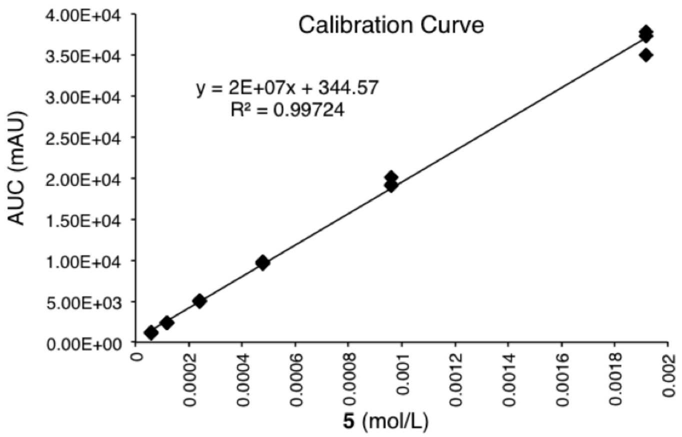

B

HPLC Injection (10 μL)	Concentration of **6** (mol/L)	AUC (mAU)
1	2.29×10^{-3}	3.52×10^{4}
2		3.56×10^{4}
1	1.15×10^{-3}	1.94×10^{4}
2		1.79×10^{4}
3		1.92×10^{4}
1	5.74×10^{-4}	9.74×10^{3}
2		9.22×10^{3}
3		9.26×10^{3}
1	2.87×10^{-4}	4.73×10^{3}
2		4.71×10^{3}
3		4.77×10^{3}
1	1.43×10^{-4}	2.28×10^{3}
2		2.34×10^{3}
3		2.37×10^{3}
1	7.17×10^{-5}	1.13×10^{3}
2		1.13×10^{3}
3		1.09×10^{3}
1	3.59×10^{-5}	5.47×10^{2}
2		5.60×10^{2}
3		5.43×10^{2}

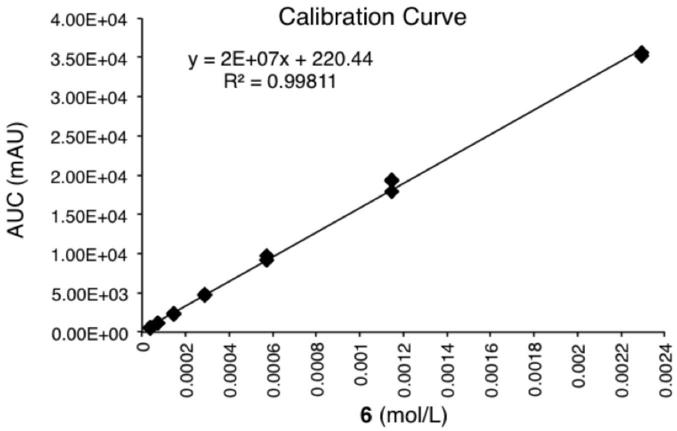

C

HPLC Injection (10 μL)	Concentration of **7** (mol/L)	AUC (mAU)
1	3.04×10^{-3}	2.77×10^{4}
2		2.82×10^{4}
1	1.52×10^{-3}	1.51×10^{4}
2		1.43×10^{4}
3		1.52×10^{4}
1	7.61×10^{-4}	7.71×10^{3}
2		7.46×10^{3}
3		7.59×10^{3}
1	3.80×10^{-4}	3.71×10^{3}
2		3.73×10^{3}
3		3.78×10^{3}
1	1.90×10^{-4}	1.76×10^{3}
2		1.80×10^{3}
3		1.84×10^{3}
1	9.51×10^{-5}	8.82×10^{2}
2		8.77×10^{2}
3		8.91×10^{2}

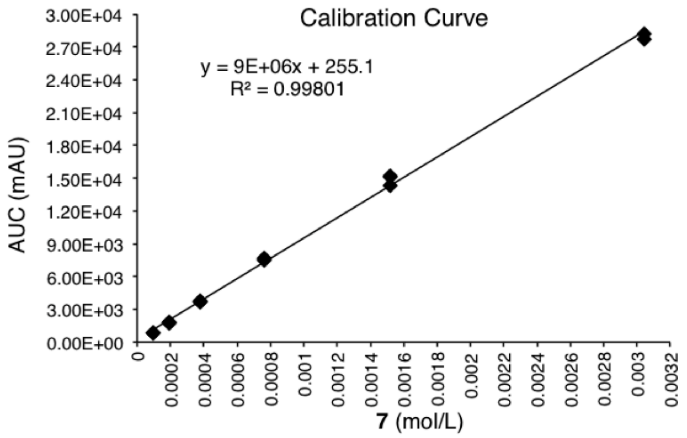

D HPLC Injection (10 μL)	Concentration of **8** (mol/L)	AUC (mAU)
1	3.14×10^{-3}	2.22×10^{4}
2		2.17×10^{4}
3		2.28×10^{4}
1	1.57×10^{-3}	1.17×10^{4}
2		1.12×10^{4}
3		1.05×10^{4}
1	7.85×10^{-4}	5.61×10^{3}
2		5.54×10^{3}
3		5.92×10^{3}
1	3.93×10^{-4}	2.89×10^{3}
2		2.87×10^{3}
3		2.97×10^{3}
1	1.96×10^{-4}	1.42×10^{3}
2		1.37×10^{3}
3		1.46×10^{3}
1	9.82×10^{-5}	7.30×10^{3}
2		9.49×10^{3}
3		7.14×10^{3}

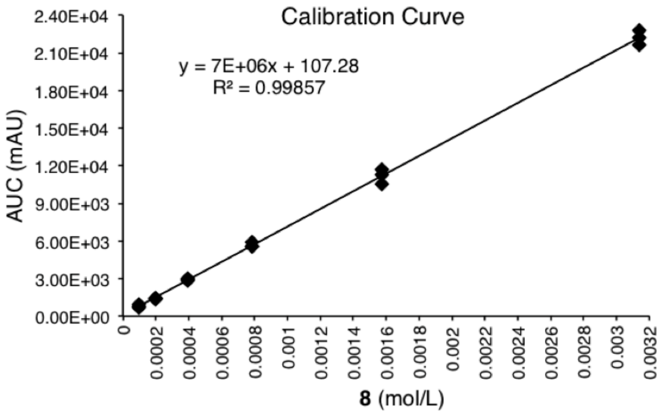

Figure S8. Determination of the calibration curves for **5**–**8**. (A) Areas under curve (AUCs) of the UV absorption and the different concentrations of pure **5** for HPLC analyses. The calibration curve and the calibration equation of **5**. (B) Areas under curve (AUCs) of the UV absorption and the different concentrations of pure **6** for HPLC analyses. The calibration curve and the calibration equation of **6**. (C) Areas under curve (AUCs) of the UV absorption and the different concentrations of pure **7** for HPLC analyses. The calibration curve and the calibration equation of **7**. (D) Areas under curve (AUCs) of the UV absorption and the different concentrations of pure **8** for HPLC analyses. The calibration curve and the calibration equation of **8**.

Table S1. Calculated physicochemical properties of **1**–**8**.

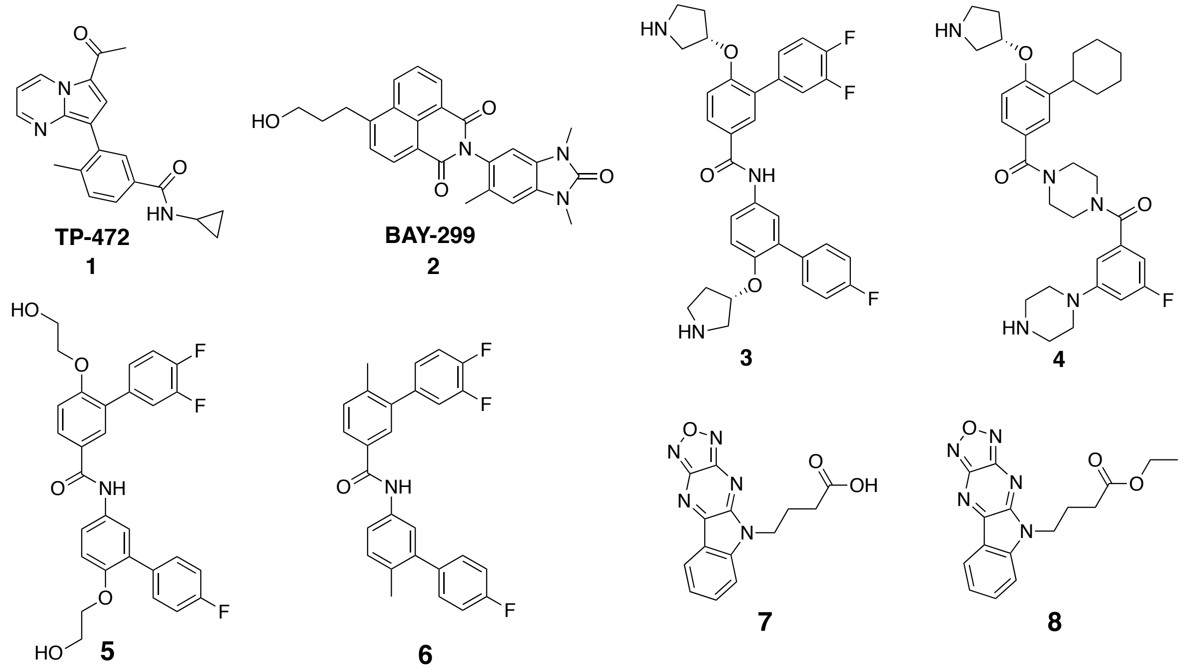

Compound	Physical Properties							
	MW[a]	HBD[b]	HBA[c]	tPSA[d]	rotatable bond	charge	cLogP	$\mathrm{logD}_{\mathrm{pH}\,=\,7.0}$[e]
**1**	333	1	3	61.8	6	0	2.48	not calculated
**2**	429	1	4	81.2	8	0	2.55	not calculated
**3**	573	3	5	71.6	8	+2	5.08	1.21
**4**	563	2	5	77.2	6	+2	3.96	0.63
**5**	523	3	5	88.0	12	0	4.14	1.70
**6**	431	1	1	29.1	4	0	7.25	7.17
**7**	297	1	7	99.2	4	–1	2.98	0.34
**8**	325	0	7	88.2	6	0	3.94	3.96

[a] molecular weight.
[b] number of hydrogen bond acceptors.
[c] number of hydrogen bond donors.
[d] topological polar surface area ($Å^2$).
[e] logD was calculated by ACD/logD.[41]

Supplementary Reference:

(41) Liao, C.; Nicklaus, M. C. Comparison of nine programs predicting pK$_a$ values of pharmaceutical substances. *J. Chem. Inf. Model.* **2009**, *49*, 2801–2812.
